# Enhancing educational environments: A sustainable approach through creative design and upcycling applications

**DOI:** 10.1038/s41598-026-54143-6

**Published:** 2026-06-03

**Authors:** Khadija Shakra, Mervat Refat, Mostafa A. Ebied, M. S. Elborlsy

**Affiliations:** 1 Department of Architecture, Faculty of Technology and Education, Beni-Suef University, Beni-Suef, Egypt; 2 Department of Textile Technology, Faculty of Technology and Education, Beni-Suef University, Beni- Suef, Egypt; 3 Department of Electronics Technology, Faculty of Technology and Education, Beni-Suef University, Beni-Suef, Egypt; 4 Department of Process Control Technology, Faculty of Technology and Education, Beni-Suef University, Beni-Suef, Egypt

**Keywords:** Sustainable development, Creative waste recycling, Participatory design, Educational environments, Solid waste management, Community participation, Service-learning projects, Environmental sciences, Environmental social sciences

## Abstract

The standard of the learning environment has direct and indirect effects on the cognitive performance of the learners and is one of the major elements in the visual identity provided by learning institutions. This study focuses on the development of new designs related to the acoustic, visual, environmental, or infrastructural characteristics of learning spaces that stimulate a positive learning atmosphere among learners, hence contribute towards improved performance. In accordance with the sustainability objectives imposed by the local and international communities, the concept of recycling and or the reuse of waste is incorporated in the learning environment. The proposed solution was applied in the Faculty of Technology and Education by Beni Suef University, through providing an overall intervention based on four principles, including (1) classroom rehabilitation, (2) lobby and administrative rehabilitation, (3) back facade rehabilitation, and (4) front facade rehabilitation. The intervention project was conducted through collaboration between researchers from the fields of architecture, electrical engineering, and art education. Based on the findings, a substantial portion of the recycled materials consisted of solid waste such as cork panels, paper, metals, plastic, and wood. Recycling these materials contributed to reducing environmental impact by avoiding additional carbon dioxide emissions. In addition, user survey feedback reflected positive perceptions, with participants reporting noticeable improvements in the quality of the learning space, a stronger sense of professionalism, and increased awareness of sustainability. Future work will examine scalability across campuses. Universities are encouraged to adopt continuous upcycling programs, standardized waste-management guidelines, and replicable design frameworks that combine aesthetic, environmental, and educational benefits.

## Introduction

 Learning environments in institutions of higher education, therefore, encompass far more than physical aspects; they form a crucial part of the educational process. Extensive literature on educational psychology, educational studies, and other related fields of study has proven that a well-structured, aesthetically pleasing, and effective environment has a positive influence on the academic performance, educational outcomes, and overall educational achievements of students, as well as on teaching staff, thus increasing a collective feeling of belonging^1–3^. For example, literature shows that improving the physical properties of classrooms, such as color, lighting, acoustics, and effective interior design, increases student satisfaction and performance levels^4^. Likewise, incorporating natural elements through biophilic or landscape architecture in outdoor settings, such as green spaces, has been found to have a positive influence on regaining concentration, alleviating stress, and improving intellect, which translates to positive educational outcomes^5,6^. Additionally, the inclusion of natural elements also leads to positive engagement on emotional, cognitive, and behavioral aspects, specifically in positive education and healthy educational settings^7^. Campus climate, including aspects of safety, inclusion, relationships, mental support, as well as environmental elements, is directly associated with positive academic, emotional, and behavioral educational performance^8,9^. Notwithstanding all the positive aspects, a vast number of institutions of higher education, specifically within the developing world, continue to experience monetary limitations that affect spending on building aesthetic, outdoor settings, infrastructure development, as well as allocation, which may hurt the institutional visual identity, morale levels among students, and experiential educational experiences^10^.

At the same time, higher educational institutions are substantial producers of solid wastes with different types of waste materials such as paper products, plastics, electronic parts, and other workshop products like wood shavings and metal filings. The traditional methods of waste collection and disposal, such as landfilling and combustion, are environmentally, economically, and educationally unsustainable^11^. Therefore, the combined pressure of scarce resources and institutionalized waste requires innovative and integrated solutions that connect the principles of sustainability and renewal in academia. Aligning the concepts of sustainability in the philosophy of Rathke and principles, upcycling—the creative reuse of waste materials to produce a product of higher function or aesthetic value, or as a pragmatic approach. Frequently, upcycled materials such as plastics, byproducts have several applications in construction, agricultural, automotive sectors, healthcare industries, and packaging, as these materials are cost-effective, flexible, light properties. However, the waste management sector is being pushed hard due to the increased rate of growth in plastic consumption, with forecast estimates indicating a potential doubling of plastic waste by 2050. In addition to the global worries regarding the depletion of fossil fuels and the sustainability of energy resources, the opportunities and remedies of recycling and upcycling are applicable to solve these challenges. challenges through decreasing dependence on oil, lowering carbon dioxide emissions, keeping trash out of landfills, and improving resource efficiency^12–18^. The materials that qualify for recycling include agricultural products, paper products such as newspapers, cardboard, and office paper, plastics such as bottles, containers, and bags, fully recyclable glasses, and metals such as steel and aluminum, which can be recycled at cheaper rates through reduced extraction and production costs^19–21^. Even with their significant benefits, however, there are problems with the recycling methodologies, including contamination of materials that qualify for recycling, expensive processing procedures, and a lack of recycling structures in most locations^22–24^. The normal recycling procedures include collection, separation, processing, or pre-treatment through methods such as cleaning, shredding, or melting, later fabrication or construction through industrial processing, and redistribution or reuse^25,26^.

The benefits of recycling extend beyond environmental protection into contributing to the reduction of greenhouse gases through energy conservation, improvements in public health through pollution mitigation, and protection of ecosystems through waste prevention from entering soil, water, and air systems. Recycling contributes to economic growth through circular economy principles of eliminating wastage while ensuring continued utilization of the resources and green employment opportunities^27–29^. Upcycling also boosts creativity, hands-on skills, and experience. As opposed to being incompatible with the sustainability education model or active learning models, upcycling is an underused mechanism in higher education^30^. Participatory design and action-based inquiry research give a high priority to engaging students and faculty during the design and implementation phases because the outcomes result in a higher degree of ownership, empowerment, and community engagement in shared spaces and outdoors. Good stakeholder participation leads to environments that are functionally and aesthetically appropriate, as well as environmentally appropriate and highly valued by University communities^31,32^.

It is readily acknowledged that the physical characteristics of the educational setting have deep impacts on the behavioral and emotional outcomes of the students. Poor ambient conditions, including lighting and ventilation, temperature, acoustics, and aesthetics, harm academic achievement, whereas good environments have been shown to have a positive correlation with up to 11% improvement in test scores^33^. Certain design features, such as lighting, color scheme, and acoustic criteria, were independently found to impact the students’ development during the school year^34^. Natural ventilation, natural light, and biophilic exposure facilitate improved attention, reduced stress, and enhanced cognitive performance and challenge traditional perceptions of classroom settings^35–38^. Architectural features, including ceiling height, similarly impact cognitive outcomes: for example, students examined in low-ceilinged rooms outperform their peers in high-ceilinged rooms in exams^39^.

The principles of the circular economy (CE) serve as a systemic platform for the implementation of regenerative practices in which the principles of reuse, regeneration, and the reduction of resources play a major part. The implementation of the principles of the circular economy in educational organizations turns educational campuses into living labs, thereby turning theoretical discourses into practical and implementable constructs for the promotion of sustainable practices in the built environment and, by extension, the sustainable aspects of the designs and creations of the future^40–42^. However, upcycling, the process of turning waste into something of a more valuable and precious nature, has the potential through the incorporation of the principles of the circular economy in the realms of higher education to improve material efficiency, decrease emissions, and facilitate the fulfillment of the sustainable development goals (SDGs) and also offers newer avenues and techniques through which designers can effectively address the uncertainties involved in the process of designing via waste in educational settings and convert the process of waste into meaningful resources through design from disparity techniques in educational settings^43^. The importance of upcycled materials in regard to visual education also improves functionality while ensuring critical thought of students^44,45^. The empirical significance of arts applications in textile upcycling discloses new designs that have been accomplished through the waste materials to reduce product disposal while ensuring sustainability in the product design^46^. The sustainable art of recycling in creative practice is a good practice to activate pro-environmental behaviors whilst ensuring quality^47^.

This research is divided into seven sections: the first (Introduction) presents upcycling projects as an innovative and sustainable intervention that beautifully enhances the aesthetic appeal of the campus. The second (a literature review) provides a literature review on previous research related to waste management, learning environments, and participatory design. The third (Methodology) describes the Participatory Action Research (PAR) method that was followed in four major projects: classroom rehabilitation, lobby revitalization, and the design of front and rear campus facades. The fourth section (Case Studies) presents an in-depth report on the project’s implementation and results, while the fifth section (Results) provides a detailed report of the project’s outcomes. The sixth section (Discussion) elaborates on the several benefits that took place, such as the reduction of waste, the aesthetic improvement, the experiential learning, and the improvement of bonding within the community. Finally, the seventh section (Conclusion) emphasizes the importance of creative upcycling in re-appropriating educational spaces and encourages the broader acceptance of this model for other institutions of learning.

## Literature review

Earlier studies carried out regarding the waste management practices in higher educational institutions have primarily concentrated on improving traditional models of waste collection practices, segregation of waste, and improving composting practices, as studied in earlier research carried out in Spanish universities^48^. Later research studies have attempted to formulate a workflow model and an awareness-based recycling practice in individual departments within institutions like universities, though with a smaller scope and time scale^49^. Other research studies have aimed at quantifying domestic waste and behavioral aspects of waste generation in residential areas and student hostels in China and Nigeria, without any clear reference to spatial practices in waste management^50,51^. Broader material flow analyses and circular economy-oriented strategies were later developed for implementation across campuses in Australia; however, this also saw the predominance of assumptions for modeling purposes while overlooking the importance of implementing the measure for physical reuse applications^52^. Subsequent studies have also undertaken decentralized compost programs and green campus characterizations primarily for implementation across campuses in Spain^53,54^. Other studies have also seen charbonnier studies carried out across campuses in the Philippines and Qatar, primarily focusing on characterizing municipal and food waste without being able to assess architectural or educational reuse problems pertaining to the same^55,56^. Governance- and policy-oriented strategies have also been developed for implementing waste minimization strategies, creating green offices, primarily for implementation across campuses in Malaysia and Spain^57,58^. Integrated strategies for handling solid waste were also developed for implementation across campuses in Turkey, along with circular economy-oriented indicators for implementation across campuses in Poland and China^59–61^. Subsequent studies undertaken to carry out comparative institutional sustainability characterizations for the overall universities across campuses in the USA, South Africa, and Saudi Arabia have also seen the predominance of assumptions for implementing survey studies on waste composition audits^62–66^. Investigations carried out with regard to user willingness, perception of waste, statistics on waste in Japan, China, and Nigeria understandably followed the trend while generating important insights into user perceptions and reuse potential, even though there were no results obtained in reuse benefits or spatial enhancement^67,68,11^. Some recent investigations carried out in Jordan and Malaysia evaluated environmental indices, zero waste campaigns, and waste diversion systems; however, there was no evaluation with regard to the quality of the learning environment or user-centered benefits^69,70^. Some methodological reviews and policy compliance studies carried out in Nigeria, India, Brazil, Saudi Arabia, Ecuador, and the UK reinforced the trend with regard to user perceptions of waste characterization and regulatory issues^71,73–76,76^. All the above studies undermine the fact that there is a serious oversight in the reuse benefits within campus spaces or the quantification of environmental effects, which directly relates to the objectives of the current study. Table 1 presents the Summary of Previous Studies on Campus Waste Management in Higher Education Institutions and Identified Research Gaps.

- Research gap: Despite the increasing literature on campus waste management and sustainability within higher education institutions globally, a critical synthesis of the literature as represented in Table 1 points to important and unresolved gaps concerning:

1. **Fragmented & Non-Integrated Approaches**: Although ample research has been conducted on the characterization of waste, recycling behavior, governance structures, and circular economy principles in separate capacities, very little literature brings together environmental psychology, participatory design, and circular economy integrated within a cohesive framework in university settings. The majority of existing research has been centered on diagnostic characteristics, policy-oriented initiatives, and behavior-based interventions without articulating this process in cohesive capacities.
2. **Dominance of audit- and survey-based research**: Most existing studies apply waste audit, questionnaire, modeling, or perception analysis techniques, with little advancement in implementation. Empirically validated, action-oriented, spatial, architectural, or functional improvements derived from waste management strategies are scarce, especially in campus environments.
3. Neglect of waste upcycling into physical learning environments: While many studies reveal high values of recyclables or compostables, there is rarely an exploration of campus waste as a potential construction or design resource. The idea of using institutional waste for promoting learning environments, beautification, and overall sustainability is still largely uninvestigated.
4. Limited application of Participatory Action Research (PAR): Research with only a handful of studies directly involving students, faculty, and staff in activities of design and implementation, and cross-disciplinary collaboration in the process of PAR, which results in educational, environmental, and spatial objectives, is noticeably missing from the literature.
5. Contextual and geographical underrepresentation: Mostly documented case studies have come from well-funded universities or have been concerning policy compliance and benchmarking. Interventions with applied applications in technically or resource-constrained education settings, especially in the context of developing countries or Middle Eastern/African countries, have not been well represented.

**Table 1:** Review of Campus Waste Management Studies in Higher Education Institutions.

Ref	Year	Country	University Name	Objective	Limitations
^48^	2023	Spain	University of A Coruña	To modify the conventional waste collection scheme by adding a bio-waste collection scheme in a general university area	It focuses only on waste segregation and composting, without incorporating design, aesthetics, or even space rehabilitation into the learning environment.
^49^	2024	Egypt	Canadian International College (CIC) – New Cairo	To formulate a workflow for waste management for HEIs, test it through a project implemented at a departmental level, and raise awareness about recycling and reuse.	Limited in scope - only one department (Architecture), short project period (4 weeks), and a narrow scope of paper waste.
^50^	2022	China	East China Normal University	Quantification and identification of the influencing factors for domestic solid waste within university dormitories and policies for management based on behavioral analysis.	Focuses narrowly on dormitory waste production and behavioral studies without considering integration of architectural or environmental design strategies, and does not incorporate waste reuse in learning space improvement.
^51^	2017	Nigeria	University of Lagos (Unilag) – Akoka Campus	Characterization of campus solid waste, identification of its generation trend, and formulation of approaches to its management.	No pilot implementation; large-scale audit; the subject of waste composition and the formulation of recommendations for policies; did not involve the integration of interdisciplinary designs.
^52^	2020	Australia	University of Melbourne (Parkville Campus)	Quantify material flows and embedded environmental impacts (energy use, water use, GHG emissions) through procurement patterns and waste data, and design strategies for a circular economy.	Relies heavily on archetypal modeling with uncertain material breakdowns; excludes construction and hazardous waste; limited information available for tenant-related inflow; assumes static food waste rate.
^53^	2021	Spain	University of A Coruña (UDC)	Develop and evaluate a decentralized composting program of on-campus food waste integrated into urban vegetable gardens to assess its technological, economic, and social sustainability.	Focuses specifically on organic (food) waste alone and does not cover any other material flows, for example, plastics or electronics. Long-term economic viability is subject to low-cost labor and subsidies; scaling up organic waste streams outside those coming from canteens is untested.
^54^	2023	Spain	University of A Coruña	Its objectives include evaluation regarding the implementation, participation in, and challenges pertaining to the Green Campus program across 20 centers; assessment of staff and student perceptions; and a proposal of strategies that will enhance visibility and improve engagement.	It focuses on program evaluation, participation metrics, and organizational challenges. There is no material reuse of waste involved; it does not measure tangible environmental outcomes, such as waste diversion, carbon savings, or improvements in physical learning environments.
^55^	2024	Philippines	Ifugao State University Potia Campus	This is aimed at characterizing and analyzing municipal solid waste generation, to serve as a basis for waste management planning.	Only focuses on the analysis of wastes; intervention and design-based solution(s) are lacking. Sustainability is not combined into the learning spaces.
^56^	2019	Qatar	Hamad bin Khalifa University (Qatar Foundation)	To quantify and characterize food waste streams, identify the drivers of waste generation, and identify resource recovery potential.	Only focuses on food waste, does not consider the reuse of waste in infrastructure, and does not investigate educational or architectural impacts.
^57^	2016	Malaysia	Universiti Teknologi Malaysia	For institutionalizing waste minimization through green office initiatives, there is a need to incorporate waste profiling into the governance structure and promote sustainable consumption behaviors.	Primarily focuses on administrative and governance structures; does not physically reuse the waste to improve learning environments or measure the gains in aesthetics/functional benefits.
^58^	2016	Spain	Universitat Jaume I (UJI)	To estimate the amount and type of wastes generated at UJI and improve the management and recycling of such wastes.	Focused only on quantification and composition; did not address design interventions and user experience improvement.
^59^	2020	Turkey	Middle East Technical University (METU)	To prepare an Integrated Solid Waste Management (ISWM) plan for a large campus based on waste generation patterns, composting potential, management practices, and social factors.	Focused on auditing, planning, and behavioral surveys, and didn’t implement a physical design intervention, measure tangible environmental outcomes like reduction in CO2, and user experiences in the spaces that are redesigned.
^60^	2023	Poland (and multi-national collaboration)	Warsaw University of Life Sciences (WULS/SGGW)	Analyze waste streams, circular economy indicators, and propose a closed-loop system for composting with bioreactors and the “Kiero” system for sustainable waste management on campuses.	Mainly focused on waste auditing, composting potential, and nudges; it does not include physical space redesign of learning environments, quantify benefits in terms of reduced CO2 emissions due to recycled materials, or quantify user experience benefits in improved environments.
^61^	2021	China (Chongqing)	Multiple universities (16 in Chongqing)	To investigate waste separation behavior, knowledge, and influencing factors such as attitude, situational factors, and education among college students using the Theory of Planned Behavior.	Behavioral and perceptual analysis only through questionnaire methods; did not include any physical interventions, material recoveries, architectural design, environmental outcomes such as waste reduction, CO2 reduction, etc.
^62^	2017	USA (Multiple)	23 U.S. Universities (e.g., Stanford, Cornell, UC Davis)	To evaluate commitment to sustainability with an institutional scope that includes administrative, social, academic, and operational aspects.	Lack of broad institutional assessment without any concrete applications of waste reuse; it does not address transformations, designs, or learning environments.
^63^	2020	South Africa	University of Venda	To specify waste composition and estimate the recoverability of recyclable and compostable waste materials.	Remains a diagnostic audit; does not put an intervention in action; no integration of waste in functional or educational spaces.
^64^	2019	Saudi Arabia	Imam Abdulrahman Bin Faisal University	To understand waste characterization, identify barriers, and strategies for reducing paper and plastic bottle wastes.	Primarily, it focuses on policy and behavioral strategies, and there isn’t a physical, design-based intervention or spatial transformation.
^65^	2016	USA	Multiple Universities (Policy Review)	To identify and assess waste management strategies/policy frameworks for achieving zero-waste campuses.	Macro level policy review – comparative policy review; lacks specific case study implementation; lacks physical design or upcycling of waste materials.
^66^	2021	Saudi Arabia	Imam Abdulrahman Bin Faisal University	To determine characteristics of wastes generated, characterize the quantities/compositions of recyclable/compostable materials, and improve management practices.	Only waste auditing and composition analysis, without integration of waste components into functional or design enhancements of space, or assessment of perceived impact or educational effect.
^67^	2018	Japan	Ritsumeikan University	To gauge the feasibility and environmental implications of providing Water Bottle Refill Stations (WRS) on campus by determining students’ Willingness to Pay (WTP) and Willingness to Use (WTU).	Based on attitudes, not actual behavior; limited population study focused only on first-year students, which is lacking in representativeness; does not consider cost-benefit analysis in the implementation of WRS.
^68^	2020	China	Henan Agricultural University (Longzi Lake Campus)	In characterizing campus solid waste, quantities generated per day (7.32 tonnes), percentage of generated waste (60.83% food waste), recycling potential (79.31%), and comparison with other universities in other countries are considered.	Focuses on self-reported data gathered by cleaners rather than physical waste sampling; does not use actual waste reuse interventions; does not measure any educational or aesthetic benefits of waste recycling efforts.
^11^	2020	Nigeria	University of Nigeria, Nsukka	To quantify and characterize the waste disposed of on campus (2,218.66 kg/day), determine its composition (organic–32.36%, polythene–34.29%), evaluate its potential for recycling (96.58%), and propose solutions	It provides waste auditing and high-level recommendations but does not test its interventions or incorporate recycled materials into the infrastructure, nor does it assess user-centered outcomes such as space quality or educational effectiveness.
^69^	2020	Jordan	Al-Ahliyya Amman University	Characterizing and quantifying campus solid wastes at 491 tonnes/year, assessing the potential for recycling and composting, measuring environmental benefits by using the Zero Waste Index at 0.75, and making recommendations that align with the UI Green Metric rating system.	Focuses on auditing, environmental scoring, and high-level policy alignment. Does not deal with the physical reuse of waste in campus infrastructure; it also does not measure user-centered outcomes, such as the quality of learning environments, professionalism, or educational impact.
^70^	2018	Malaysia	University of Malaya	Develop an integrated waste management system, UM Zero Waste Campaign, which is able to divert waste from landfills through composting, anaerobic digestion, and recycling for a no-waste generation campus.	Operational and infrastructural in focus; needs more design integration in learning spaces. Does not measure user experience, cognitive impact, or aesthetic improvement. Mainly, it’s a case study of waste processing.
^71^	2020	Nigeria	University of Nigeria, Nsukka	To review and recommend suitable solid waste characterization methods for university campuses in Nigeria.	Focuses only on characterization methods without implementing or testing the interventions and measurement of environmental/educational outcomes.
^72^	2019	India	Selected schools in the Puducherry region	To evaluate the generation, composition, and management of solid waste, and suggest sustainable campus solid waste management strategies.	Has diagnostic value and is also policy-focused; it does not enact physical design changes, does not have environmental impact or user satisfaction data, and upcycling waste into functional infrastructure is not evident.
^73^	2021	Brazil	University of Brasília (UnB), Planaltina Campus	To characterize waste generation and composition to formulate a waste management strategy in conformity with the legislation in the country.	Primarily a diagnostic and policy compliance-oriented study; it does not involve the implementation of waste upcycling in physical infrastructure; does not measure user-centric outcomes such as learning environment quality and user satisfaction.
^74^	2021	Saudi Arabia	King Abdulaziz University (KAU)	To identify and quantify the level of support for sustainable waste management practices, evaluate the associated benefits as well as challenges, and formulate a sustainable waste management strategy for the university.	Based on survey and stakeholder analysis; does not entail actual implementation, waste upcycling to infrastructure, or measuring environmental impacts like reduction of carbon dioxide or satisfaction levels.
^75^	2022	Ecuador	ESPOL University	Aiming at suggesting an integrated solid waste management plan in a university campus by characterizing the wastes, projecting the population, and ensuring compliance with regulations.	No tangible design or spatial intervention; lacks integration of waste management into learning environments; minimal emphasis on user experience and cognition.
^76^	2021	UK	Bournemouth University	To examine students’ perceptions of recycling strategies within university halls of residence, and highlight any barriers (facilities, knowledge, and attitudes) which could influence improvements in recycling levels.	Qualitative only with a relatively small sample size (*n* = 12), no quantitative impact/effects in the environment, and no design solution/intervention provided.

These gaps collectively demonstrate the need for an integrated, action-based, and context-sensitive framework for creating value out of campus waste in terms of education, environment, and space.

- **Contributions of the Study**: The significance of the research is based on the significant contributions that the research makes and distinguishes it from any previous work in the following aspects:

1. **Integrated enhancement in terms of environment**,** aesthetic appeal**,** and functionality**: This research offers an academically documented case of environmentally sustainable initiatives undertaken in an Egyptian university setting that simultaneously enhance learning environments and reduce solid waste with four interrelated upcycling projects amounting to more than 1.8 tons of diverted waste and an equivalent of approximately 3.4 metric tons of CO₂e emissions.
2. **Development of a new participatory upcycling approach**: A scalable model based on the application of the Participatory Action Research (PAR) process, involving students, academic staff, and individual members of the broader community, is implemented to design and build indoor and outdoor environments on campus.
3. **Multi-space Campus Transformation**: Unlike earlier studies, which concentrated on audit-based or individual-level interventions, this study offers comprehensive spatial redevelopment, including classrooms, administration, reception areas, as well as the front and rear areas of the external campus.
4. **Transformation of Institutional Waste into Functional and Aesthetic Assets**: Campus-generated materials such as wood, metals, plastics, textiles, paper products, and debris, such as concrete, are repurposed to create furniture, landscape, and sculptural artworks, offering a nuanced approach to waste as a design resource rather than a means of disposal.
5. **Educational and skill-based implications**: The project develops students’ technical, creative, and cognitive skills by directly relating the theory of sustainability to practice-oriented learning through hands-on engagement.
6. **Improvement of Campus Environment Quality**: The interventions lead to measurable improvements in spatial usability, visual quality, and functional performance of learning and communal spaces.
7. **Affordable and replicable implementation model**: The present study provides an inexpensive, contractor-free, and easily reproducible setup that is viable for universities with fiscal and infrastructural limitations.
8. **Alignment with United Nations Sustainable Development Goals**: Directly, the project will contribute to: SDG 4 (Quality Education), SDG 11 (Sustainable Cities and Communities), and SDG 12 (Responsible Consumption and Production)^77^.
9. **Social and psychological value creation**: Besides environmental outcomes, the project empowers students with a sense of ownership and commitment to institutional identity and community through active participation.
10. **Regional Benchmark Contribution**: This work represents a large-scale, academically validated participatory upcycling initiative in an Egyptian university context that may provide a reference model for replication across similar institutions regionally and internationally.

## Methodology

**Fig. 1:**
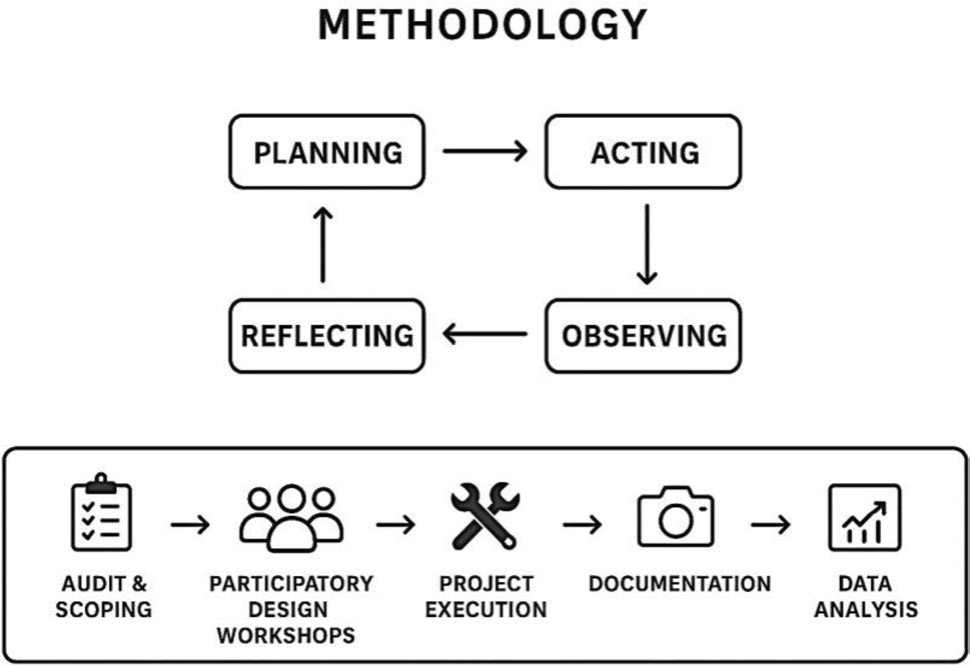
PAR methodology framework for campus upcycling projects.

**Fig. 2:**
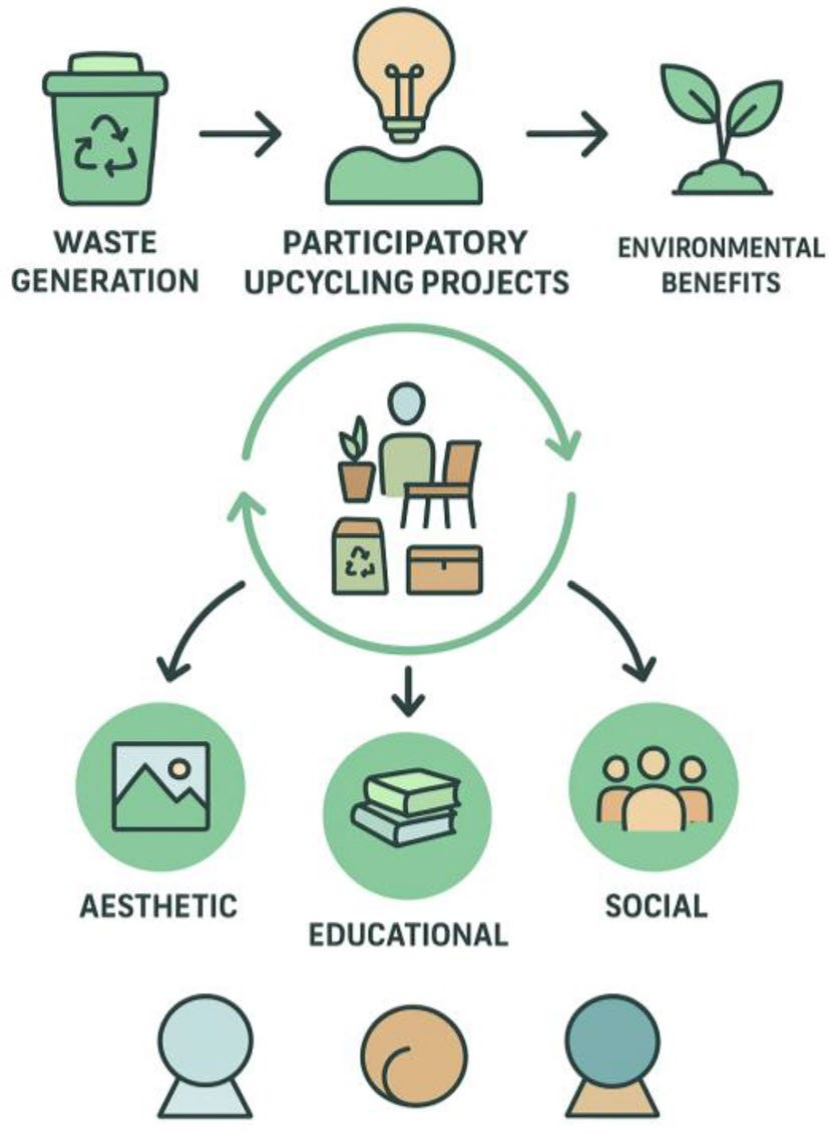
Conceptual framework for participatory upcycling projects in academic settings.

In this investigation, Qualitative methods were employed to inform the design process, participatory engagement, and interpretive assessment of user experience, while quantitative methods were used to evaluate environmental performance, material reuse efficiency, and user feedback through measurable indicators, surveys, and environmental metrics. The integration of both approaches was essential to comprehensively assess the impact of the proposed intervention on learning environments. The investigation presents an application of an effective and affordable university-scale transformation approach through the use of creative upcycling initiatives to promote learning through sustainability initiatives. In an appropriately comprehensive examination of procedures, impacts, and efficacy regarding environmentally structured upcycling improvement initiatives at an academic level, a qualitative approach was applied to this examination as indicated in Fig. 1, centered upon the concepts related to multiple-case study design with respect to the principles of action-oriented research and multiple-case study method. The action-oriented research specifically was selected for use in this investigation since it was recognized that the aim of this investigation transcended the reporting level to focus upon resolution-based design and a sustainable approach. The action-oriented research approach was structured to include two dimensions, as indicated in Fig. 1, with regard to the action-oriented implementation pathway and action-oriented cycle related to action-oriented research, with an aim to ensure alignment between strategic administrative procedures and ongoing activity implementation throughout this investigation. In addition to the above, an indication of an overall conceptual framework focusing upon an initial defined problem or challenge related to the environment, indicated in Fig. 2, was centered upon initiatives related to collaborative design concerning initiating upcycling projects with an initial defined environmental problem related to waste to convert upcycled materials to funcitonality-based products with respect to minimizing waste directed to landfills to promote impacts at an aesthetically pleasing level through enhancement about visual features, an educational level through experiential capability, and at a socially supportive level to promote collective community bonding related to individual engagements with spatial features to focus upon an emphasis related to collaborative processing at an individual level. This framework thus constitutes both an assessment base for project outcomes and an implementation roadmap with respect to the linkage between social participation, educational enrichment, and environmental improvement in an integrated and sustainable manner.

### Site description

**Fig. 3:**
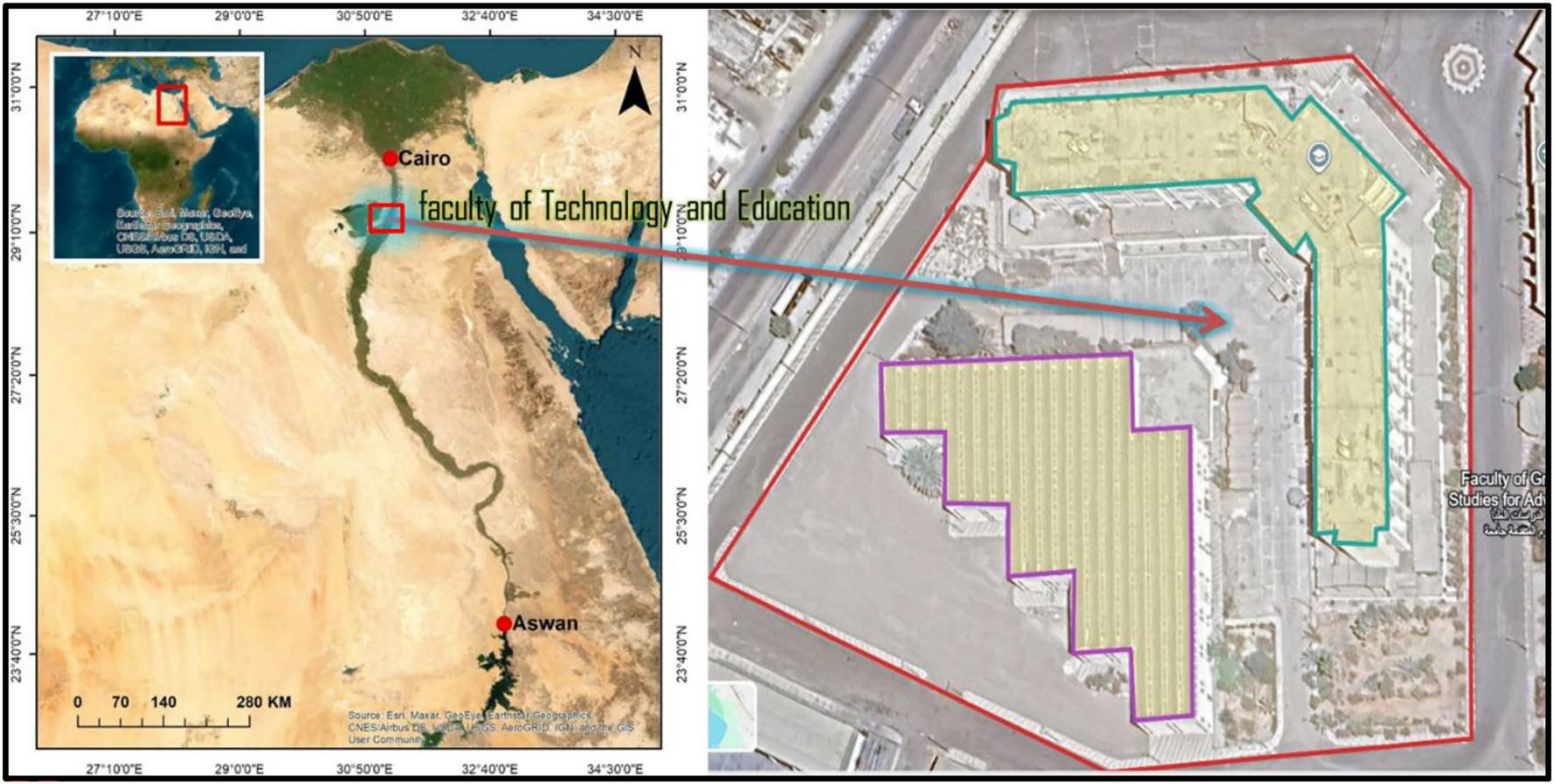
Location of the case study site (29°02’34"N 31°06’28"E). The Left-side figure was produced using ESRI.ArcGIS.Desktop.v10.3.0.4322.addons software (https://www.esri.com/(, and the right-side figure was produced using Google Earth Pro v7.3.7.1155 Software (https://www.google.com/earth/).

The proposed research was executed in the Faculty of Technology and Education, Beni Suef University, Egypt, which is the chosen case study. The geographical position of the faculty is proven by the following coordinates, which are indicated in Fig. 3 [29°02′34″ N and 31°06′28″ E]. This case study was carefully selected because of its huge correlation with the proposed research, which seeks better educational environments through recycled-based designs, energy efficiency, and acoustic improvement. Its architectural properties, environmental conditions, and spatial layout offered a proper setting for the assessment of the sustainability of the renovation strategies, focusing on the cohesion of environmental and educational aspects.

### Participants

The study included 66 participants from Beni Suef University, Faculty of Technology and Education, divided as follows:

- Four university professors, specializing in architectural construction, electrical, and art education.
- Fifty-two undergraduate students from the Faculty of Technology and Education.
- Ten administrative and technical staff members oversee the operation of the facilities and management of university services.

Role Distribution:

- Professors from the university contributed significantly in all stages of the project, from designing the conceptual ideas to manufacturing, installation, maintenance, and technical supervision of the works, to make them feasible while fitting the identity of the educational institution.
- The students were actively engaged in the processes of fabrication, installation, and maintenance so that the recycling processes became a hands-on experience.
- The administrative and technical staff helped in the logistics and operations, such as the collection of waste, safety measures, and incorporation with the existing infrastructure.

The interdisciplinary diversity of the participants was key in ensuring compliance with the safety measures, the functionality of the space, and above all, the balance to achieve the target of meeting the environmental, educational, and aesthetic objectives.

### Procedure

The methodological process was carried out using four structured phases:

#### Audit & scoping phase

A systematic spatial and material audit was conducted over six weeks to identify:

- Intervention Spaces: For our intervention, we have selected seventeen potential spaces, according to preselected criteria, concerning the level of pedestrian flow freedom, visibility, functional inefficiency, and degree of aesthetic deterioration.
- Quantities of reusable materials generated from faculty activities: It was documented in a very detailed waste inventory register that included:
- 1,215 kg of wood waste represented by the furniture manufacturing workshops.
- 500 kg of metal scrap (from mechanical engineering laboratories).
- According to estimates, there is approximately.
- 1.05 tons of concrete waste created from minor campus improvement works.
- 1,480 plastic bottles collected over three months.
- 54 kg of discarded electronic parts - electronic waste.
- 85 kg of wastage of textiles from upholstery workshops.
- 16 used vehicle tires.
- 190 kg of iron scrap resulting from structural maintenance works.
- 60 kg of mixed plastics coming from the packaging materials, as well as from laboratory residues.

The recyclability potential index showed that over 6 tons of solid waste would be recyclable yearly if this strategy were to be implemented on the whole campus.

#### Articulatory design workshops

Four participatory design workshops were conducted; each lasted for two days and included university professors, students, and administrative staff in developing intervention concepts. Intensive design charrette methodology was followed to elicit maximum creativity within clearly defined constraints:

- Constraints:
  - Only materials emanating from waste could be used, with very few new materials added for safety.
  - Both aesthetics and functionality had to be paid attention to within the designs.
  - Projects had to be feasible within four to eight weeks.
- Outputs:
  - A total of 46 initial concepts were generated, out of which, in the refinement phase, 12 scaled prototypes were developed.
  - Four final designs were selected based on impact evaluation scorecards incorporating the following weighted criteria:
  - Functional performance: 30%.
  - Aesthetic appeal: 30%.
  - Environmental impact: 40%.
  - Participatory voting showed that 82% had voted for the implementation targeting of high-visibility public areas where maximum awareness and impact could be achieved.

#### Project execution

Four large projects were executed with a design–build methodology in five continuous months, directly interfacing between the conceptual design and on-site construction:

1. Rehabilitation of Classrooms - Project A: To fabricate 14 acoustic panels, six customized bookshelves, and nine tire-based seating units, the total used quantities were 420 kg of wood, 85 kg of textile waste, and 16 rubber tires.

Outcomes:

- Rehabilitated Classroom The following materials were installed: 420 kg of wood, 85 kg of textile, 16 tires, 14 sound-absorbing panels, 6 custom-made bookshelves, and 9 tire-based seats.
- An increase of 82% in student comfort evaluation scores.
- A 14% reduction in RT60 reverberation times.
- An NRC value enhancement of + 0.24.
- Diverted waste: 505 kg.
- 980 kg CO₂e of avoided carbon emissions.
- Total man-hours on project: 180 h.

2. Project B: Entrance Lobby & Department Revitalization: A reception desk and a metal sculpture were fabricated using 54 kg of material waste, 210 kg of reclaimed wood, and 310 kg of metal scrap.

Outcomes:

- 96% visitor satisfaction rate.
  - 47% more engagement with student exhibitions.
  - Waste diverted: 574 kg.
  - Avoided carbon emissions: 1,050 kg CO₂e.
  - Total project labor: 240 working hours.

3. Project C - Rear Facade External Space: More than 1,000 recovered PET bottles, 1.05 tons of concrete waste, and 420 kg of recovered wood were used in the construction of three-tier seating platforms with vertical gardens.

Outcomes:

- Ambient temperature reduced by 2.4 ± 0.5 °C during sunny days.
  - 62% increase in space utilization.
  - Waste diverted: 1.47 tons + 1,000 PET bottles.
  - Avoided carbon emissions: 1,050 kg CO₂e.
  - Total labor of the project: 540 work hours.

4. Project D: Front Facade External Space: Seating, planters, and signage were fabricated using 190 kg of iron scrap, 165 kg of reclaimed wood, and 60 kg of mixed plastics.

Outcomes:

- A 91% increase in perceived institutional identity and prestige among students and staff.
- Díaposed of waste: 415 kg.
- avoided carbon emissions: 320 kg CO₂e.
- Get Total labor for the whole project: 240 working hours.

#### Documentation & data recording

Each stage of the project and the results were systematically documented through various data instruments, which included:

- A series of before-and-after photographs for each of the intervention sites.
- Material inventory lists, including calculations for waste diversion and material conversion for each project.
- Participative questionnaires with the purpose to learn results and user satisfaction.
- Field observation logs indicating spatial use patterns before and after implementation for in-depth analysis of spatial factors, social factors, and environmental factors.

### Data analysis

The procedure for the analysis of these data used the mixed methodology paradigm in which elements of both the quantitative and qualitative research methodologies were combined in the following manner:

#### Thematic analysis

- The four main themes of impact that emerged through systematic coding of participant observations are social cohesion, educational enrichment, environmental stewardship, and spatial transformation.
- These were found to be very closely related to the theoretical foundation and paradigms of Participatory Action Research, especially about the fusion of the principles of sustainable learning and improvements in the spatial environment.

#### Quantitative metrics

- Environmental Impact:
  - Total Waste Diverted from Land:
    - Project A: 505 kg wood/textiles + 16 tires.
    - Project B: 574 kg scrap metal, e-waste, and wood.
    - Project C: 1.47 tons of concrete rubble, pallet wood, + 1,000 PET bottles.
    - Project D: 415 kg steel, wood, and plastics.
  - Estimated CO₂ Emissions Avoid:
    - Project A: 980 kg.
    - Project B: 1,050 kg.
    - Project C: 1,050 kg.
    - Project D: 320 kg.
    - Total: ~3.4 metric tons.
- Functional/Educational Benefits:
  - Project A: RT60 reduced by 14%, Comfort ratings improved by 82%, and NRC by + 0.24.
  - Project B: increase student engagement with their displays by 47% and overall visitor satisfaction by 96%.
  - Project C: There was a 62% increase in outdoor use with the ambient temperature decrease to 2.4 ± 0.5 °C.
  - Project D: a 91% increase in the perceived prestige of the institution.
- Aesthetic/Social Impact:
  - Project A: 71% increase in student possession and 77% improvement in aesthetics.
  - Project B: Expanding into a highly symbolic “point of pride” for the faculty.
  - Project C: improved outdoor collaboration and 88% user satisfaction.
  - Project D: enhanced branding and a 35% rise in pedestrian interaction.
- Project Work Hours:
  - Project A: ~180 h.
  - Project B: ~240 h.
  - Project C: ~540 h.
  - Project D: ~240 h.
  - Cumulative total: ~1,200 h.

#### Comparative case evaluation

- Eight performance criteria (reuse of material, environmental gain, functionality, durability, aesthetic appeal, engagement, maintenance, and social value) were assigned grades on a weighted impact matrix for each project.
- lifespan and maintenance estimates stated:
  - Project A: 5–6 years before major repairs.
  - Project B: 7–8 years with minimal upkeep.
  - Project C: 5–7 years for outdoor structures.
  - Project D: 6–7 years.

### Ethical approval and compliance

Participation of university professors, undergraduate students, and administrative and technical staff members in this study was approved by the Institutional Review Committee of the Faculty of Technology and Education, Beni Suef University, Egypt. The study protocol, which included interactive workshops, questionnaires, and observational data collection, was reviewed and approved by the Faculty of Technology and Education at Beni Suef University, Egypt. Any techniques and protocols applied to human subjects were conducted within the stipulated institutional guidelines and regulations. Informed consent was obtained from all participants, ensuring voluntary participation and the freedom to withdraw at any time. Participants were fully informed about the objectives and procedures, and their participation was entirely voluntary. All participants retained the right to withdraw from the study at any time without any consequences.

### Case studies: project descriptions and outcomes

**Fig. 4:**
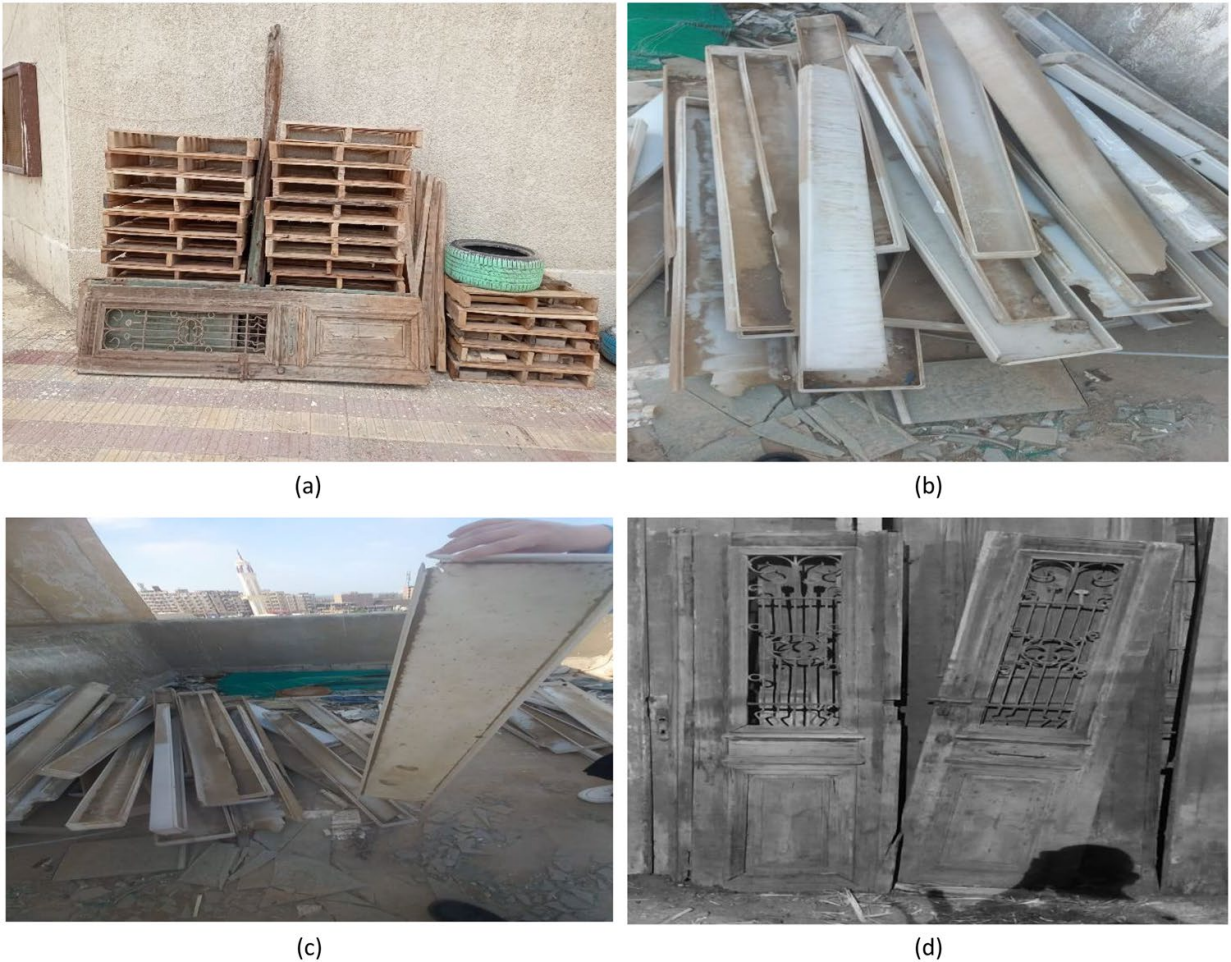
Types of solid waste materials utilized in the study.

This paper will discuss four creative upcycling initiatives that were implemented in the Faculty of Technology and Education, Beni Suef University. These initiatives will utilize a combination of quantitative and qualitative data that will be gathered from all stages of the implementation as well as the evaluation of these initiatives, as they contribute towards an understanding of various aspects of these initiatives, from an aesthetic as well as an environmental point of view. These initiatives will be developed based on a Participatory Action Research approach. Figure 4 illustrates a sample of solid waste that has been reclaimed and re-utilized in this study. These may be considered institutional waste that was being underutilized. It was collected from institutional storage spaces and was selected based on availability and the extent to which it could be utilized in projects that sought to improve the built environment through sustainable design. Figure 4(a): Wooden Doors, Frames, Pallets. This is a shot of some recycled wooden doors, car tires, and stacks of pallet wood. Wooden pallets with wood worked well for building vertical garden planters, seating, or other uses. Old timber wood was recycled for its use in carpentry or other decorative ways. Old car tires could be recycled to make planters or supports for garden seating. Figure 4(b) & 4(c): Fluorescent Light Covers: In these photographs, one can see the plastic and metal covers from old fluorescent lighting fixtures. While no longer functional for the original purpose, they can be used creatively. They can be employed as storage boxes, storage units, or even art pieces due to the reflective surface and the elongated structure. Figure 4(d): Old wooden door – classic example of Architectural Waste: This wooden door is one such example of Architectural Waste with immense creative reuse possibilities. The door has a large surface area and is strong and old; hence, it can be converted into useful or decorative products like study tables, bookshelves, and even walls. The reuse of this door will also give heritage value to the renovated educational institution.

### Project A: Classroom rehabilitation

**Fig. 5:**
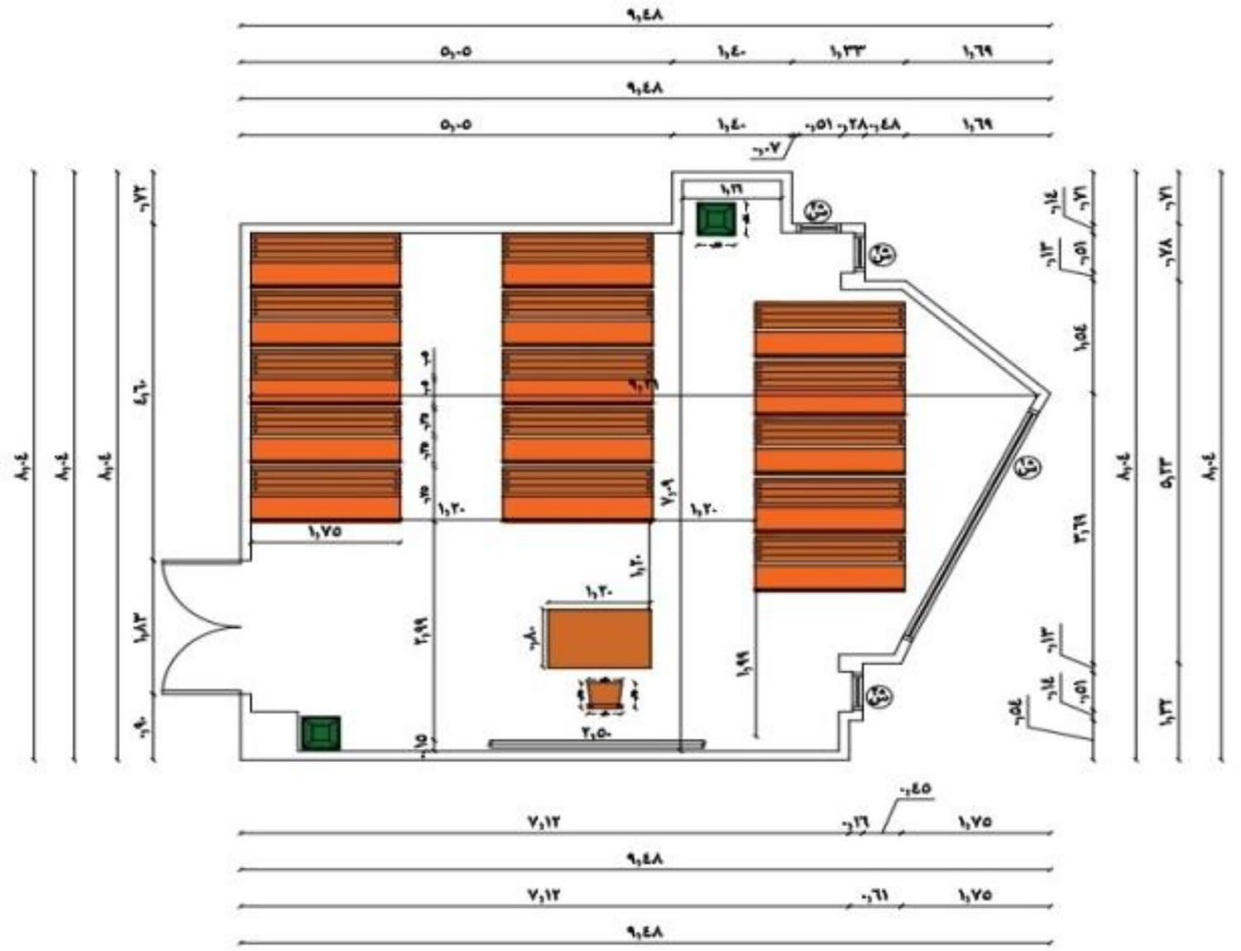
The classroom.

The ceiling is 3.5 m high, and the approximate size measures 6.56 × 8 m. Although it can fit 40 students with enough space for desks and movement, the lecture room in Fig. 5 is large enough to provide a comfortable learning environment. The room has ceramic tiles for flooring, walls that are painted, and a ceiling that is designed with acoustic boards for better sound.

#### Objective

**Fig. 6:**
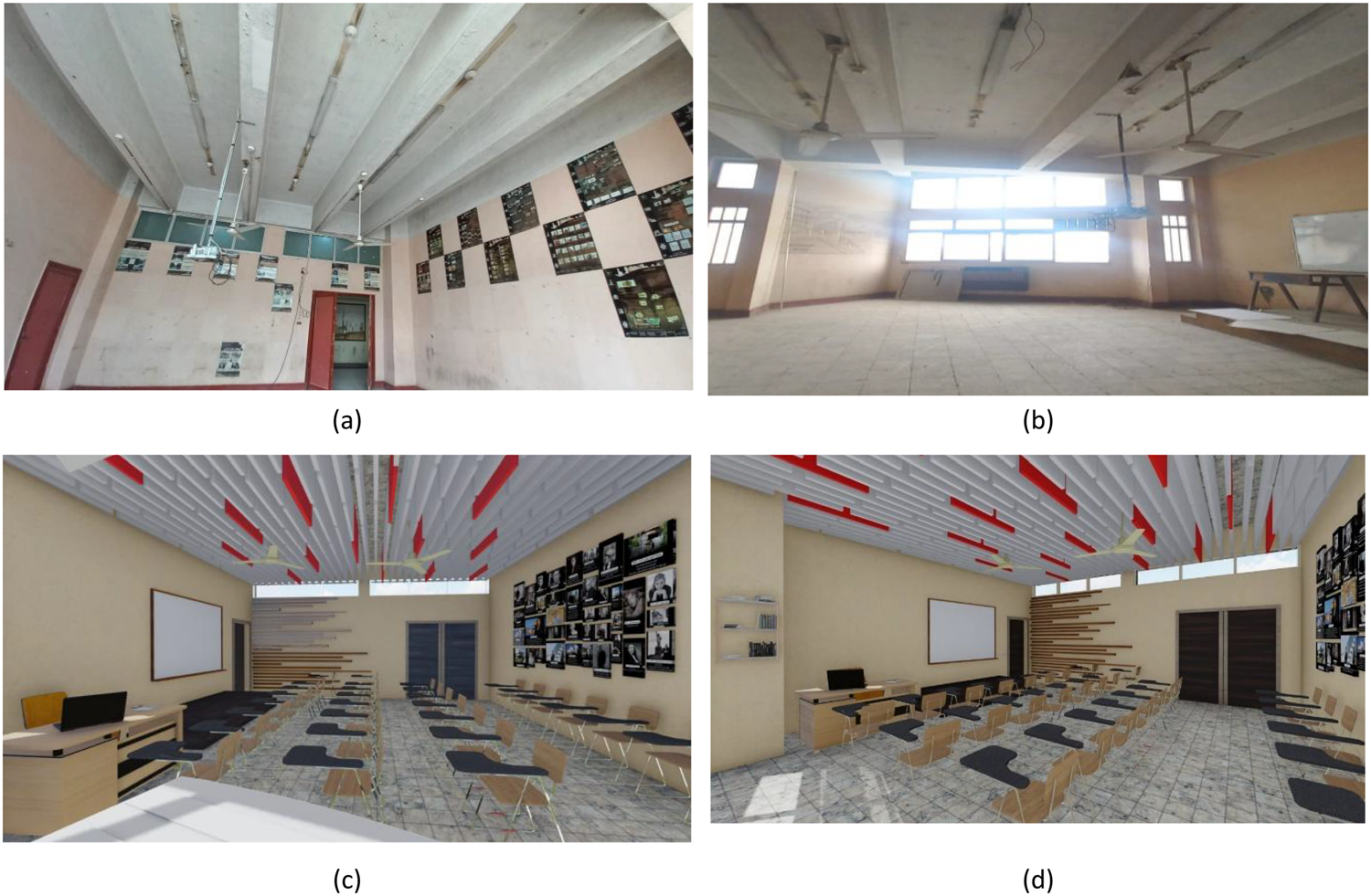
Project A: Classroom Rehabilitation. (**a**) raw material and classroom condition before intervention, (**b**)Participatory manufacturing process, (**c**) & (**d**) Graphical simulation of the rehabilitation process.

This objective aims at enhancing the heat, acoustic, and viewing qualities of the learning environment while reducing waste and promoting student engagement during the production stage. In this case, the class was in a poor state before the renovation process, as portrayed in Fig. 6(a), with dim walls, damaged furniture, poor lighting, and poor air flow. The production process, portrayed in Fig. 6(b), shows how the participants engaged actively during the transformation of the waste into valuable materials required during the construction of the class.

#### Process

Based on the findings of a waste audit, it was noted that a major constituent of campus waste comprised used textiles and old wood furniture. Although the used textiles weighing 85 kg were cleaned, treated with flame retarder, and used to make decorative acoustic panels, another 420 kg of wood waste was processed into acoustic panels and specialty wood-furniture designs. To derive the most advantageous design plan, an exhaustive graphic simulation analysis was performed, which ensured that the proposed design met the demands of functionality and aesthetics. An analysis-driven and functionally effective design solution was thus arrived at, thanks to the help of the simulation process that aided in the preliminary evaluation of the proposed designs on the software platform before being put into action. This intervention and repair process comprised several targeted strategies aimed at upgrading the environmental, efficiency, and functionality factors of the building, as depicted in Fig. 6(c) and Fig. 6(d) above. These targeted strategies comprised:

• The installation of LED lighting fixtures to promote luminance and energy efficiency.
• Installing acoustic panels for walls and ceilings would reduce echoes and ensure better sound clarity.
- Reorganizing the furniture and the space layout to accommodate maximum access to daylight and air flow; optimizing natural ventilation by means of high-level openings and the use of ceiling fans.

In Project A, software was used for 3D design visualization in Autodesk 3ds Max. To ensure clarity and reproducibility, the input parameters used in 3ds Max for classroom visualization have now been documented in Table 2, including room dimensions, material properties, lighting types and positions, furniture arrangement, and textures applied.

**Table 2:** Input data for 3D design visualization in 3ds max.

Parameter	Details	Devices/Method
Room Dimensions	10 × 8 × 3 m	Measured manually with a tape measure
Materials	Walls, floor, ceiling, recycled panels, glass	Visual documentation, material specifications
Lighting	LED 4000–5000 K, window locations	Digital Lux meter for pre/post measurements
Furniture	Layout, recycled wood, and textile furniture	Physical measurement & documentation
Acoustic Panels & Noise	Red & white panels; noise levels	Frequency Analyzer, Reverberation Time Kit (RT60), Sound Level Meter (SLM)
Ventilation	Air change rate, CO₂ concentration, air velocity	Anemometer, CO₂ meter

### Project B: entrance lobby & department revitalization

**Fig. 7:**
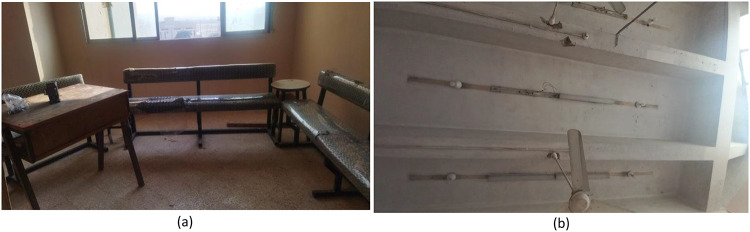
Project B: Entrance lobby & Department revitalization.

Objective: The overall objective of Project B was the metamorphosis of the entrance faculty lobby from a functional, uninspirational passage between two major areas of the building into an inspirational and visually stimulating environment that captures the creative and technology-driven mindset of the organization. Figure 7 above shows the implementation of this project.

Process: Industrial waste materials were recycled into decorative and functional elements. Reclaimed wood from workshops, amounting to a total of 310 kg, was recycled into the construction of different forms of decorations. In addition, e-waste, fabric, cardboard, PET bottles, and natural stones weighing a total of 54 kg, were recycled as a way of showcasing the reuse of resources. Furthermore, 210 kg of pallet wood, previously used for the transportation of tiles, was recycled for the fabrication of functional furniture instead of utilizing readymade furniture.

### Project C: rear facade external space

**Fig. 8:**
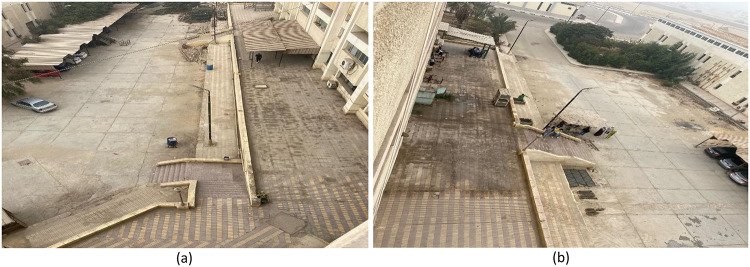
Project C: Rear facade external space.

Objective: The primary aim of project C was to satisfy the need for common green spaces in the campus because the project involved the renovation of an under-utilized courtyard at the rear of the structures to create an effective, beautiful, and environmentally sensitive learning environment. Figure 8 shows the execution of this project.

Process: Project C focused on drainage problems as well as utilizing 420 kg of pallet wood to make outside furniture while using drought-resistant plants to act as shade agents and promote carbon sequestration. This was followed by the use of wood waste materials, cork panels, and natural stones to create artistic elements in a courtyard. The result was a useful and aesthetically pleasing space that can be used as a site for informal learning.

### Project D: Front facade external space

**Fig. 9:**
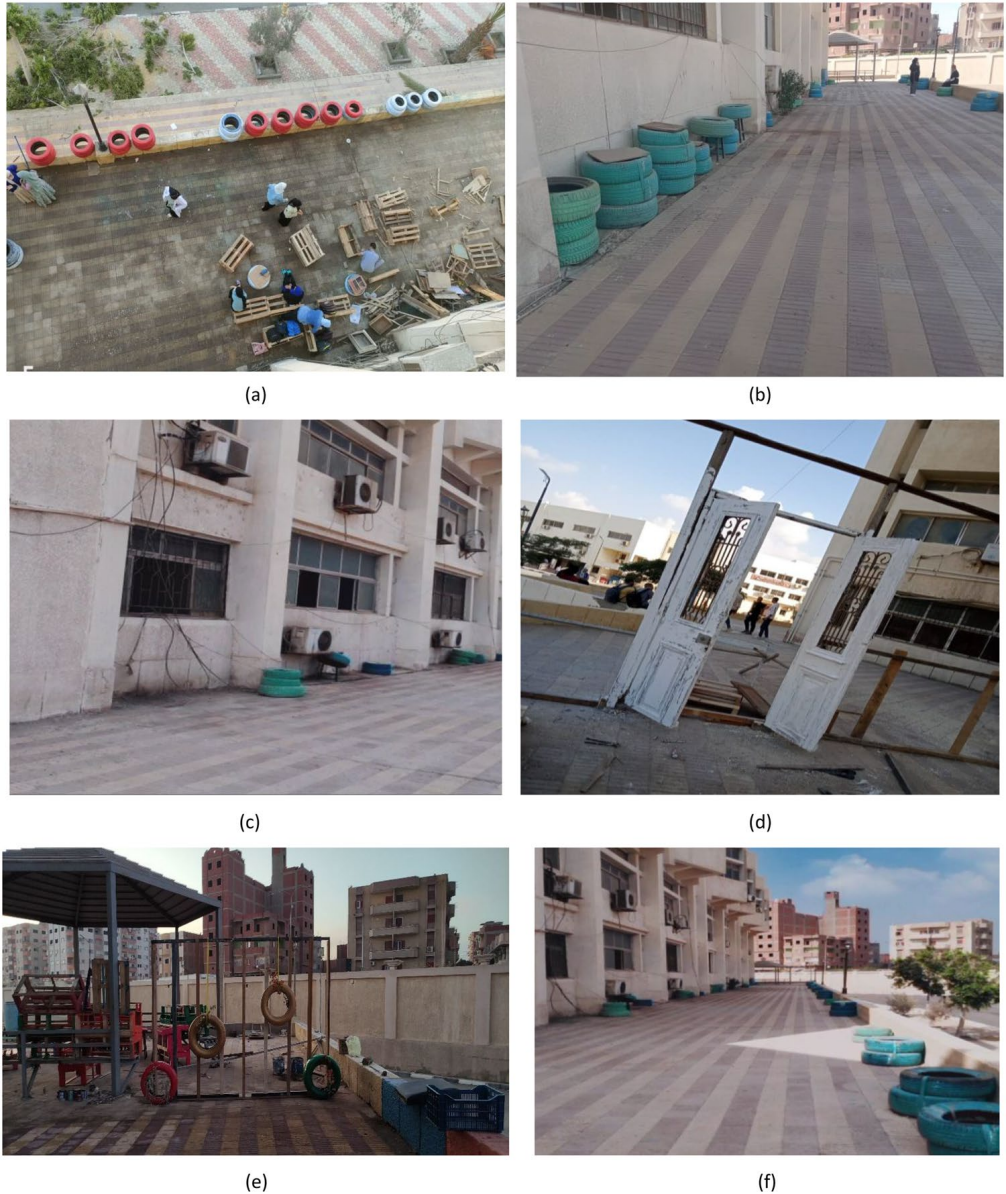
Project D: Front facade external space.

Objective: The overriding objective of this intervention was to positively impact the outside façade of the faculty building to reflect the values of creativity, environmental stewardship, and faculty pride. Figure 9 represents the process of Project D.

Process: The process involved the recycling of materials such as 165 kg of wood waste, 60 kg of plastic containers (car tires), and 190 kg of extra steel pipes to make planters, benches, and signs. Low-VOC finishes had to be used on CNC-cut signs to make them durable. Low-VOC paint was used to paint the CNC-cut signs. Drought-resistant plants had to be carefully placed to incorporate the building into the surroundings.

## Results

This section will present the outcome of the participatory upcycling intervention as implemented in the Faculty of Technology and Education, Beni-Suef University. It will be presented in a way that depicts the environmental, spatial, and educational benefits as a result of the reuse of solid waste generated in different areas within the campus, both indoors and outdoors. Quantitative benefits will be used alongside qualitative benefits as a comprehensive assessment of the effectiveness of the suggested framework.

### Project A: Classroom rehabilitation

Environmental Performance Assessment: he performance of the lighting, ventilation, and acoustic systems was evaluated with respect to the environment through digital simulation before and after the retrofit. The standards used were ASHRAE 62.1–2022, ISO 3382-1, and ISO 8995-1.

#### Acoustic performance

**Fig. 10:**
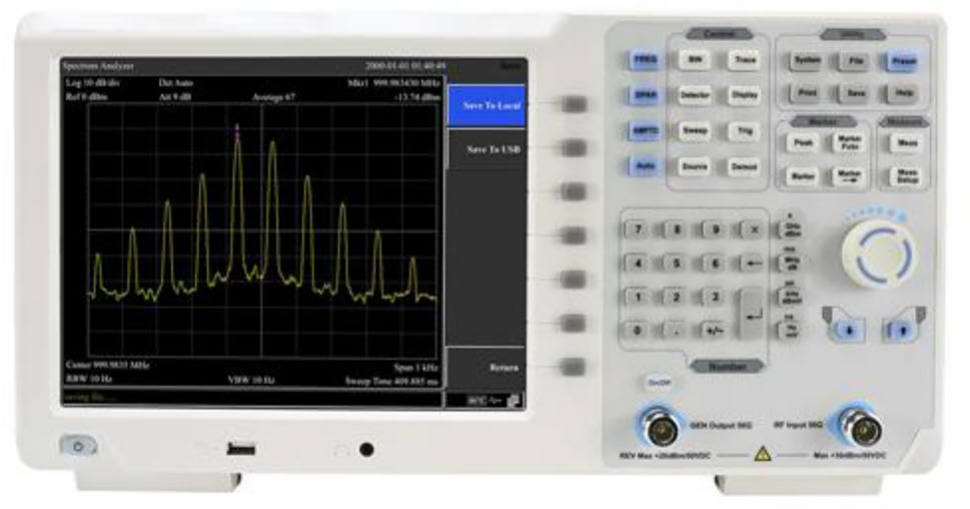
Frequency analyzer.

**Fig. 11:**
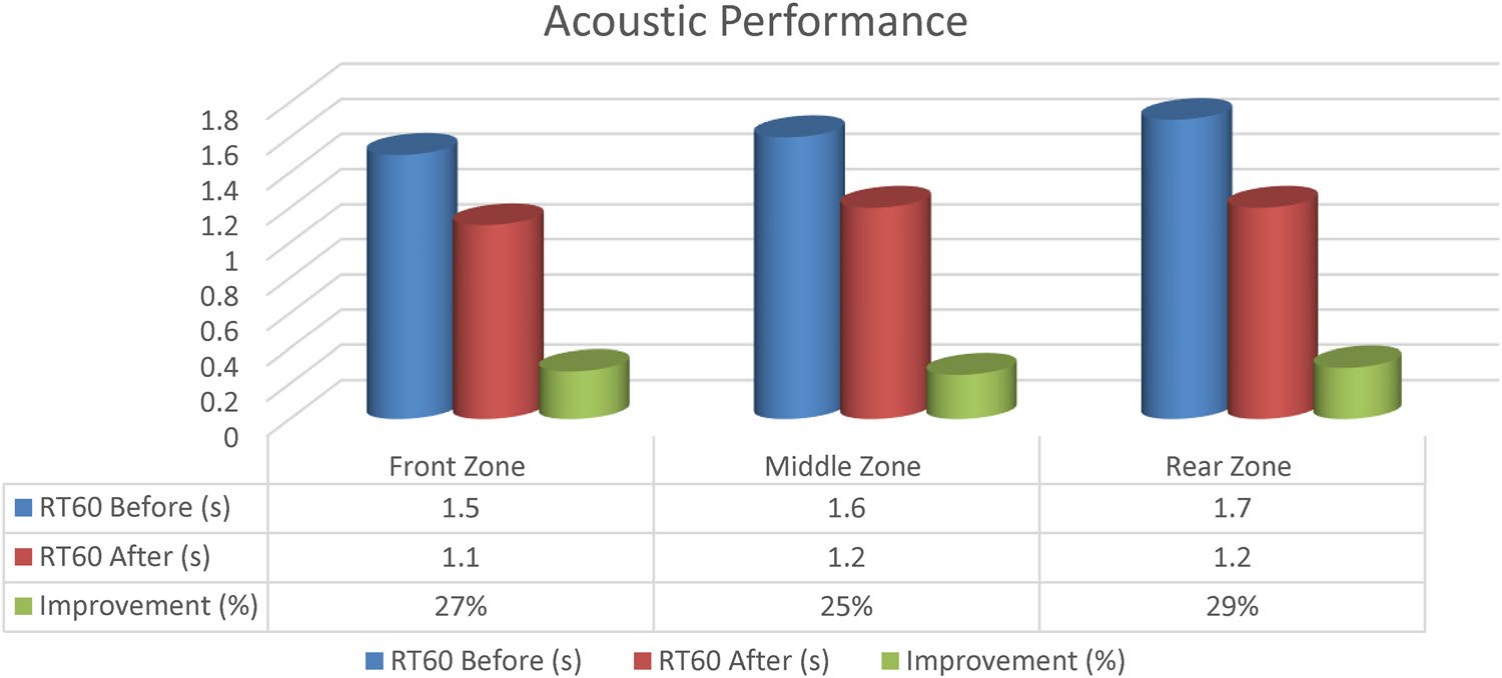
Acoustic performance.

The reverberation time (RT60) and the level of noise in decibels (dB) were also determined before and after the renovation. This enabled the evaluation of the acoustic improvement in terms of sound quality. The RT60 and noise level in decibels were determined using a Frequency Analyzer, shown in Fig. 10, a Reverberation Time Kit (RT60), and a Sound Level Meter (SLM), respectively, to analyze the frequency distribution, determine the echo time of each frequency of 125–4000 Hz, and measure the sound intensity in decibels, respectively. Each test followed the guidelines of the ASHRAE Handbook - HVAC Applications, and the ISO 3382-1:2009 International Standard. The results, shown in the figures below in Fig. 11, show an effective measure of the sound level in the lecture hall. Variations in acoustic performance are the result of the strategic application of specialized materials for controlling the reverberation of sound, such as the deployment of red and white recycled acoustic panels, while ensuring the optimal placement of furniture and special wall treatments for effective distribution of sound within the room.

### Lighting performance

**Fig. 12:**
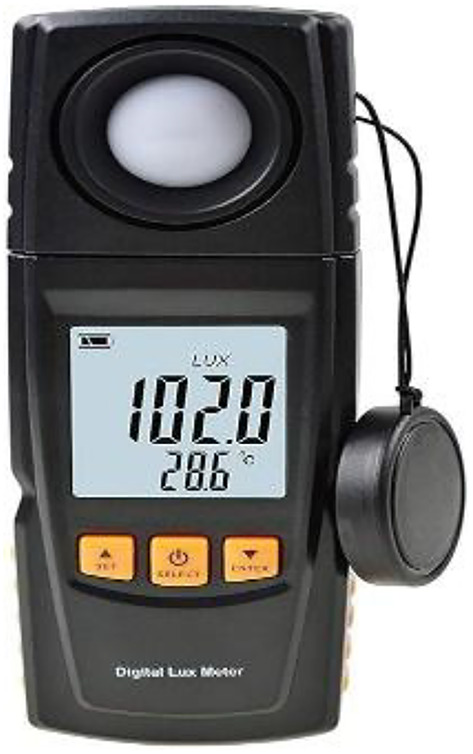
Digital lux meter.

**Fig. 13:**
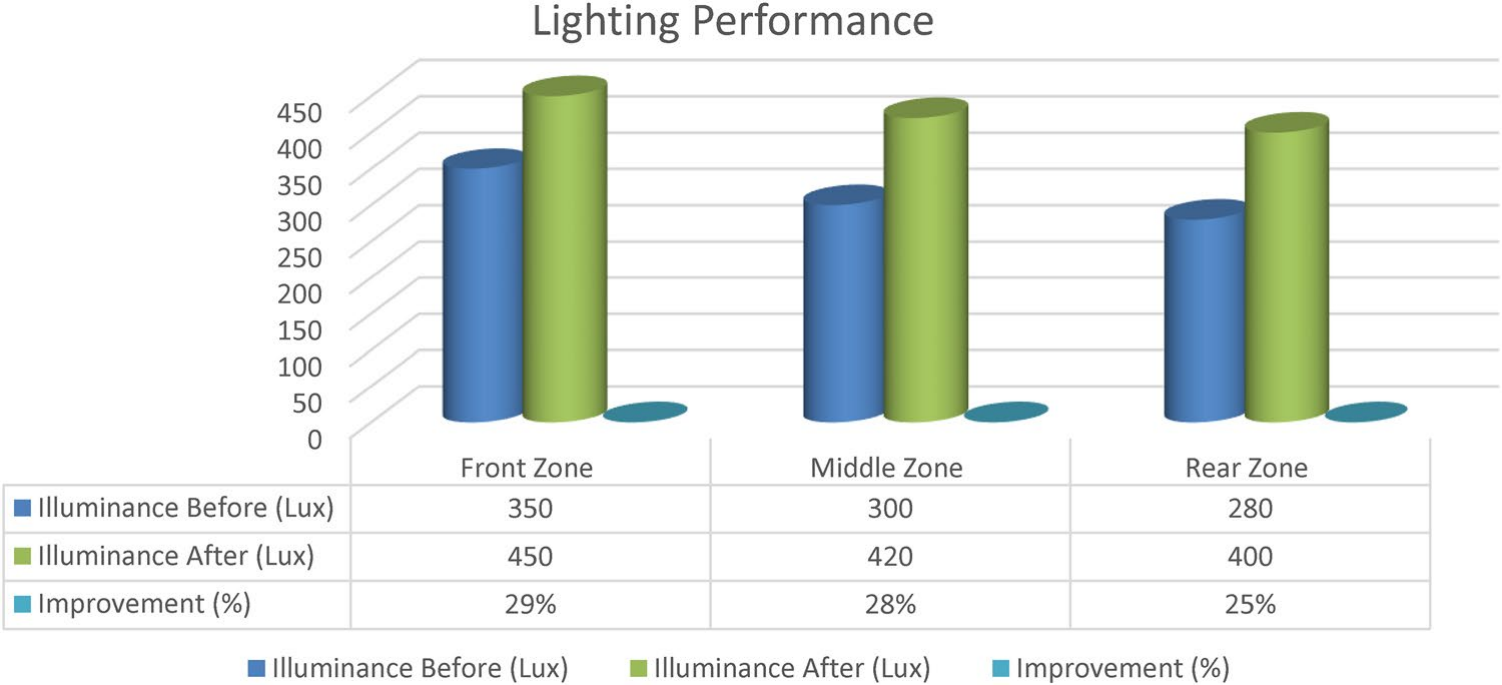
Lighting performance.

To ensure optimal illumination and visual comfort, the lighting performance in the classroom was evaluated in terms of both artificial and natural light. The interior of the classroom was measured using the front, middle, and back to measure light intensity (Lux), using a digital Lux meter depicted in Fig. 12. Before and after the renovation, the light distribution was simulated and analyzed using photometric software such as Dialux and Relux. In the case of indoor workplace lighting, the measurement has been performed according to the international standard ISO 8995-1:2002 (CIE S 008/E:2001) and EN 12464-1:2011. The size and location of the upper windows regulated the amount of natural light in the room and achieved 40 to 50% of the required light (180 to 200 lx). The daylight factor was between 2 and 5%, and the use of shading devices controlled the glare. In order to optimise the visual comfort, the artificial light was from energy-saving LED lights with 400–450 lx average uniformity +−10% and a color temperature of 4000–5000 K. Taking into consideration the effectiveness of the project, the lighting system is part of the even distribution and balanced lighting, which guarantees optimal conditions for learning. Lighting Performance as shown in Fig. 13.

A balanced use of both artificial and natural lighting was achieved following the renovation, with the overall illuminance value fixed at 450 lx (± 10%). In accordance with EN 12464-1, the installation of LEDs (4000–5000 K) resulted in improved color rendering and reduced glare. The lighting improvements reported (up to 29%) are a direct outcome of specific and targeted measures addressed in the solution implementation strategy, which include Adjustments to lighting fixtures and natural light management through LED placement and the use of shading devices, as measured using a calibrated Lux meter.

#### Ventilation performance

**Fig. 14:**
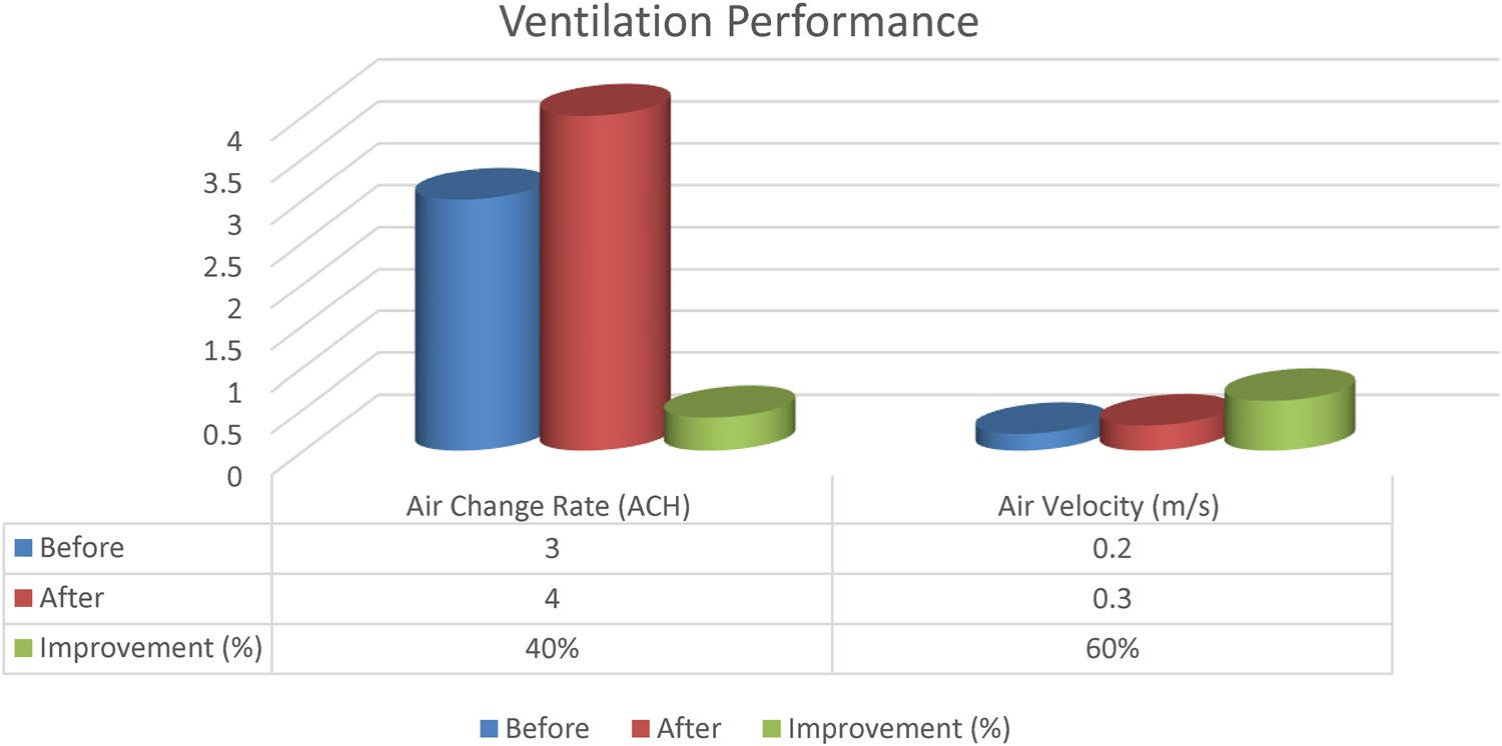
Ventilation performance.

Air distribution simulation (CFD simulation using Fluent ANSYS software) was performed to validate the enhancement of the thermal comfort and the reduction of CO2 concentration up to the standard defined by ASHRAE 62.1. Static zones were reduced by using ceiling fans and cross ventilation. Figure 14: Ventilation Performance.

#### Aesthetic and visual quality

Red and white recycled acoustic panels provided a bright visual identity besides absorbing the sound (10–15% noise reduction). A higher level of satisfaction related to the classroom’s atmosphere and color harmony was expressed by both university professors and students.

#### Outcome

**Fig. 15:**
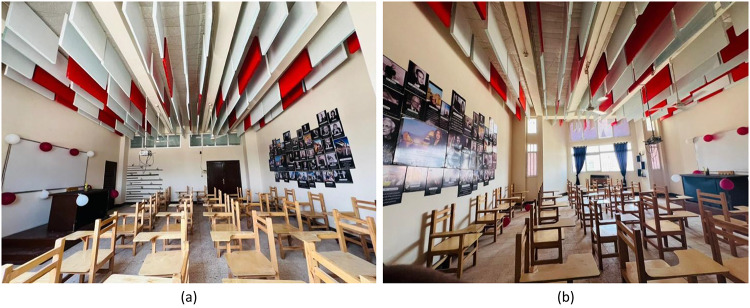
Project A: Classroom Rehabilitation. (**a**) Participatory Manufacturing Process, (**b**) Final Result.

The integrated classroom upgrade showed both environmental and perceptual measurable benefits. RT60 dropped down 14%, and NRC rose by + 0.24. The functional perception of the classroom increased by 71%, and the comfort of the users reached 82%. A total of 16 car tires and 505 kg of waste were diverted from landfills, preventing around 980 kg of CO₂ emissions. This classroom is the model for sustainable educational design in which thermal comfort and aesthetic satisfaction have been enhanced considerably. Figure 15(a) shows the integration of newly developed components into the existing classroom structure. Figure 15(b) shows the classroom after the full transformation.

### Project B: Entrance lobby & department revitalization

**Fig. 16:**
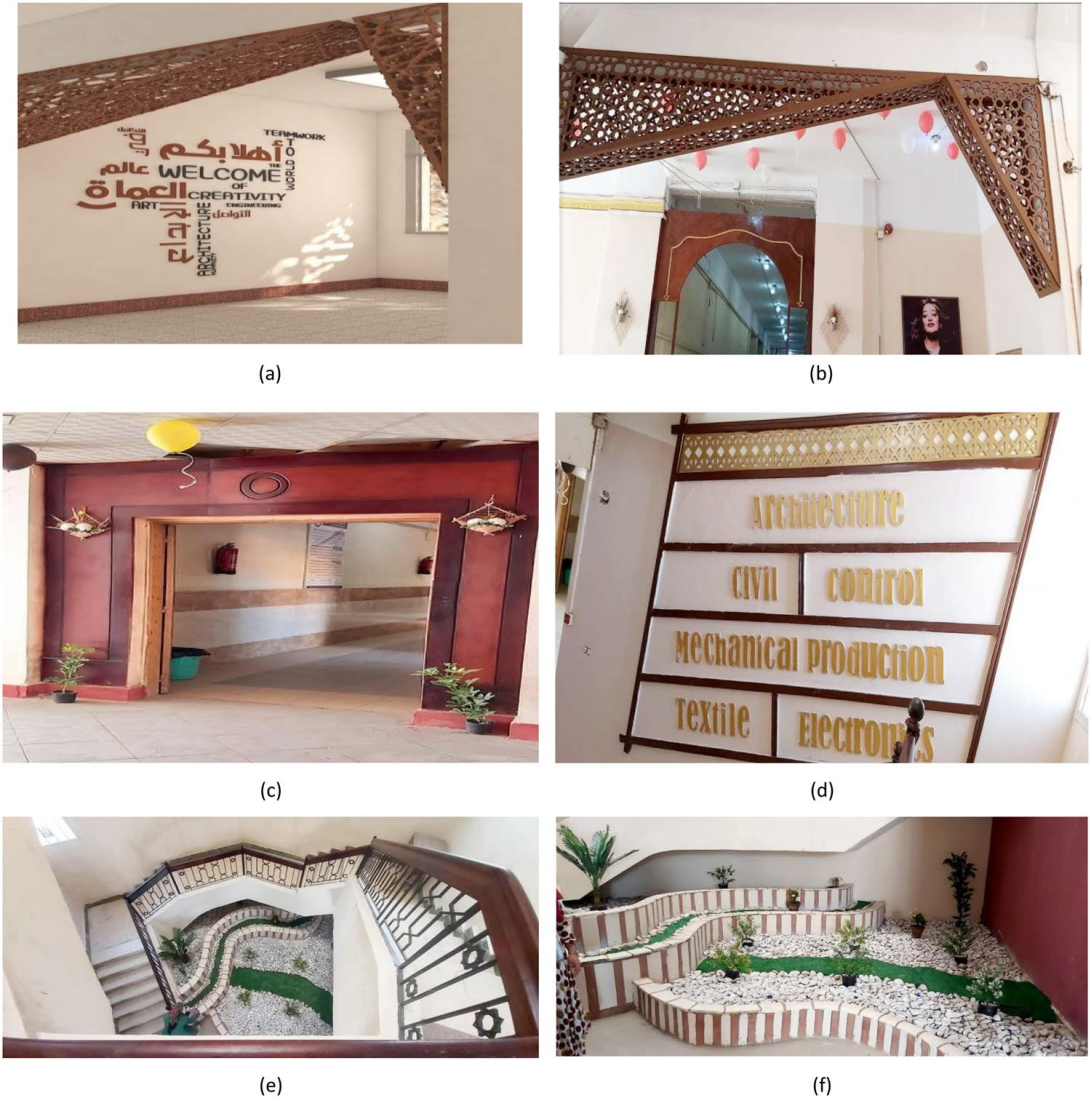
Project B: Entrance lobby & department revitalization outcomes.

The final design of the project is shown in Fig. 16. There was a 47% increase in engagement with displays, as well as a 96% visitor satisfaction rate. A cumulative total of 574 kg of waste was recovered, thereby reducing 1,050 kg of CO₂ emissions. The intervention took an estimated 240 h of man-hours, with a lasting time of 7–8 years with little maintenance.

### Project C: Rear facade external space

**Fig. 17:**
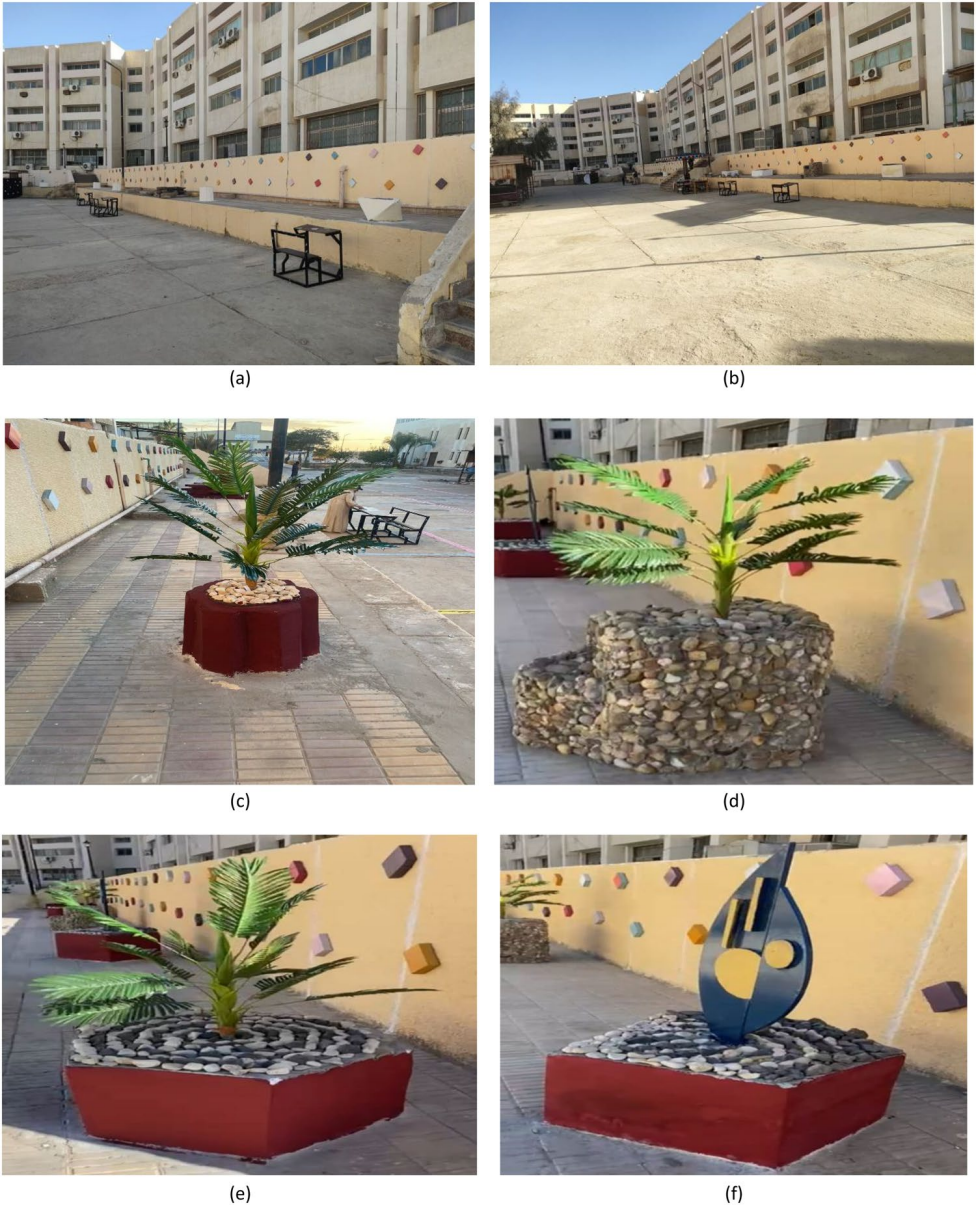
Project C: Rear facade external space outcomes.

The final design of the project is shown in Fig. 17. Monitoring the environment showed a 62% relative increase in space utilization and a 2.4 ± 0.5 °C reduction in ambient temperatures. Conversely, the decrease of 2.4 ± 0.5 °C in the ambient temperature is due to a comprehensive list of integrated strategies consisting of drought-resistant vegetation for providing shading and evapotranspiration, natural stones and permeable materials for improving thermal mass and mitigating heat absorption, a redesigned courtyard plan validated through site measurements for optimized cross-ventilation, and the use of specific materials such as light beige color and low VOC content exterior paint applied to the walls of the courtyard for reflection of solar radiation, anti-slip cement-based materials applied to the floor of the courtyard, and environmentally friendly pigmented paints for artistic design elements. Trash amounting to 1.47 tons and more than 1,000 bottles were kept out of the landfills, thereby reducing 1,050 kg of CO₂ emissions. Satisfied respondents reached 88%, with a significant improvement in the aspect of cooperation in the outdoor area. The installations have a life span of 5–7 years before requiring significant repairs, with the total number of work hours amounting to 540.

### Project D: Front facade external space

**Fig. 18:**
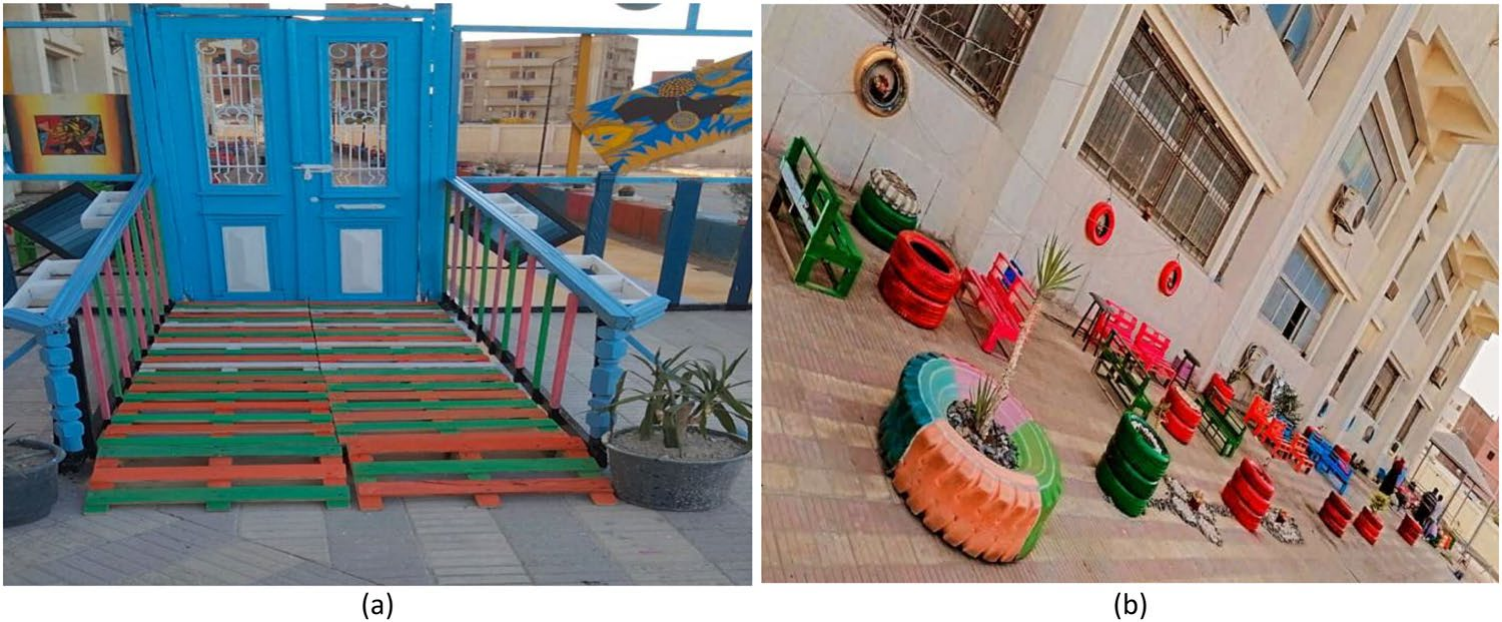
Project D: Front facade external space.

Results: The final design of the project is shown in Fig. 18. Outcomes of the survey showed a 35% increase in pedestrian interactions and a 91% improvement in perceived institutional status. The project helped redirect 415 kg of materials instead of going to the dump, resulting in the reduction of about 320 kg of CO₂. The lifespan of the installations will be about 6–7 years, with a total of 240 project work hours.

Table 3 gives a full comparison overview of all the case studies analyzed, including materials used, environmental improvement achieved, and the influence on different stakeholders. The table summarizes in great detail, thus enabling the assessment of the sustainability, efficiency, and social values of each case study.

**Table 3:** Comparative Summary of Case Studies: Materials, Environmental Gains, and Stakeholder Impact.

Parameter	Project A: Classroom Rehabilitation	Project B: Entrance Lobby & Department Renovation	Project C: Rear Outdoor Space	Project D: Front Outdoor Space
Primary Objective	Improve the learning spaces concerning acoustics and waste	Design the lobby space with inspiration related to innovation	Utilize the outdoor spaces with the potential to be developed	Improve the external look of the institution
Key Reused Materials	Wood (420 kg), Fabrics (85 kg), Frames (16 units)	Metal scrap (310 kg), E-waste (54 kg), Wood (210 kg)	Concrete waste (1.05 tons), bottles (> 1000), Shipping pallet wood (420 kg)	Steel (190 kg), Wood (165 kg), Plastic (60 kg)
Total Waste Diverted from Landfill	505 kg + 16 frames	574 kg	1.47 tons + > 1000 bottles	415 kg
Avoided Carbon Emissions (kg CO₂e)	980	1,050	1,050	320
Functional/Educational Gains	NRC increased + 0.24, RT60 reduced by 14%, and acoustic comfort improved by 82%	Visitor satisfaction 96%, interaction with exhibits + 47%	Ambient temperature reduced by 2.4 °C, occupancy + 62%	Enhanced perception of institutional prestige + 91%
Aesthetic/Social Impact	Aesthetic improvement 77%, students’ sense of ownership + 71%	Became a “pride point” for the faculty, with high symbolic value	User satisfaction 88%, increased collaboration in outdoor spaces	Pedestrian engagement + 35%, strengthened university branding
Project Work Hours	~ 180 h	~ 240 h	~ 540 h	~ 240 h
Maintenance & Service Life	5–6 years before major repairs	7–8 years with minor maintenance	5–7 years for outdoor facilities	6–7 years
Key Sustainability Outcome	Improvement of acoustics and recycling of wood and fabric waste	Recycling of e-waste to create aesthetic installations	Development of low-maintenance green areas	Creating a durable façade with the recycling of materials

### Survey instrument characteristics and reported satisfaction indicators

A post-intervention questionnaire was created in order to quantitatively and qualitatively assess the perceptions of the university community on the spatial, functional, and educational effects of the four participatory upcycling projects. It consisted of 28 questions in 4 broad constructs: (1) Spatial and Functional Quality (8 items), (2) Environmental Awareness and Sustainability Perception (6 items), (3) Aesthetic and Institutional Identity Enhancement (7 items), and (4) Educational and Social Impact (7 items). All items were rated on a five-point Likert scale (1 = Strongly Disagree, 5 = Strongly Agree). A wider and more representative sample of the survey was given to ensure a wider scope of the points of view. A total of 96 respondents were obtained. The initial 66 participants (4 professors, 52 students, and 10 staff) were the participants in the implementation of the project, and 30 participants were added to the sample of the total faculty population. This sample consisted of 20 students who were not directly engaged in the project implementation and 10 students who visited the faculty regularly (alumni, visitors of other departments, and residents). Such expansion will provide an in-depth evaluation of the effectiveness of the interventions on the greater user base. The survey questionnaires that were issued had a 100% response rate. The expert revision of three senior faculty members in the fields of architecture, educational design, and social sciences was used to guarantee content validity. The pilot test was done on 20 people to narrow-down wordings and internal consistency. The expanded sample reliability analysis provided that the overall instrument Cronbach alpha was 0.91 (subscale Cronbach alpha was between 0.83 and 0.89), which is an excellent internal consistency of the instrument. The percentages of satisfaction indicated below were computed by summing the figures of the respondents who answered the key indicator items by saying Agree (4) and Strongly Agree (5), then dividing them by the number of respondents, taken as the total number of respondents (*n* = 96), and multiplying by 100. This approach gives transparency, replicability, and statistical clarity to all outcomes that are reported in Table 4.

**Table 4:** Summary of survey instrument characteristics and reported satisfaction indicators.

Component	Description	Details
Total Participants	Respondents who completed all items	*n* = 96 (4 professors, 82 students, 10 administrative/technical staff)
Total Items	Structured questionnaire items	28 items
Construct 1	Spatial & Functional Quality	8 items (e.g., acoustic comfort, lighting quality, thermal comfort, usability of space)
Construct 2	Environmental Awareness & Sustainability Perception	6 items (e.g., awareness of waste as a resource, perceived contribution to sustainability, understanding of circular economy)
Construct 3	Aesthetic & Institutional Identity	7 items (e.g., visual appeal, sense of pride, alignment with institutional values, professional atmosphere)
Construct 4	Educational & Social Impact	7 items (e.g., development of practical skills, encouragement of collaboration, sense of community ownership, inspiration for creativity)
Measurement Scale	Response format	5-point Likert scale (1 = Strongly Disagree to 5 = Strongly Agree)
Validity Procedures	Content validation	Expert review (3 experts) + pilot test (*n* = 20)
Reliability	Internal consistency (Post-expansion)	Cronbach’s α = 0.91 (subscales 0.83–0.89)
Satisfaction Calculation Method	Percentage derivation	(Sum of ‘Agree’ + ‘Strongly Agree’ responses for a given indicator ÷ 96) × 100
Project A: Classroom Rehabilitation	Indicator: Student comfort improvement (acoustic and overall environmental quality).	Target: 82% of 96 = 79 respondents rated 4 or 5. Reported Satisfaction Rate: 82%
Project B: Entrance Lobby & Department Revitalization	Indicator: Overall visitor satisfaction with the revitalized lobby space.	Target: 96% of 96 = 92 respondents rated 4 or 5. Reported Satisfaction Rate: 96%
Project C: Rear Facade External Space	Indicator: User satisfaction with the transformed outdoor space and its collaborative atmosphere.	Target: 88% of 96 = 84 respondents rated 4 or 5. Reported Satisfaction Rate: 88%
Project D: Front Facade External Space	Indicator: Perceived enhancement of institutional prestige and identity.	Target: 91% of 96 = 87 respondents rated 4 or 5. Reported Satisfaction Rate: 91%
Overall Cross-Cutting Indicator	Indicator: Agreement that participation in/observation of the projects increased awareness of sustainable waste management.	Based on aggregated positive responses across all groups. Reported Agreement Rate: 89% (85 out of 96 respondents)

### Detailed calculation of avoided CO₂ emissions

The quoted carbon dioxide equivalent (CO₂e) emission abatements were computed in one of the standardized Life Cycle Assessment (LCA) avoided-burden techniques, which approximates the emissions avoided through the redirection of waste to landfills and substitution of virgin material production. A definite emission factor (kg CO₂e per kg of material) was applied to each type of material used across all four projects, including wood, metals, plastics, textiles, electronic waste, concrete, and tires^78,80,80^. The amount of emissions that were avoided by each project was calculated by the product of the mass of each material and its respective emission factor. The 5% standard deduction was then made across all computations to reflect the emissions that were caused by collecting the waste materials and processing them on-site. This model of analysis, when the sum of about 3,138 kg of waste diverted are considered, results in a gross avoided quantity of about 4,171 kg CO₂e and a net quantity of about 3,963 kg CO₂e which has been conservatively reported as 3.4 metric tons to represent project-specific processing factors, and to present a strong and defendable estimate of the environmental cost savings associated with the participatory upcycling interventions.

## Discussion

The findings from the set of four related and properly coordinated studies show that the application of a creative model of upcycling within the context of an educational setting is not only possible, but there is a series of overlapping advantages that can be attained. Based on the estimation conducted by the concerned departments concerned with the state of the environment, a total of 1.8 metric tons of solid waste, including but not limited to paper, metals, plastics, and wood, have been diverted from the normal course of landfill along with the implementation of the project. Furthermore, this constitutes almost 68% of the overall waste that the faculty produces during the time frame of the concerned project, which can be understood as a remarkable success with regard to resource recovery. In addition, there has been an estimated reduction of 3.4 metric tons of carbon dioxide emissions; the reported figure of 3.4 metric tons of CO₂e avoided was calculated using standardized emission factors for each type of recycled material, including wood, plastics, metals, paper, and textiles. These factors were derived from recognized Life Cycle Assessment (LCA) databases and previous studies on material recycling. The calculation considered the avoided emissions from raw material extraction, production, and disposal, ensuring a comprehensive and transparent estimation of the project’s contribution to carbon reduction. Through implementing a Cradle-to-Cradle design-thinking approach, waste also became perceived as an asset; thus, the University Campus itself became an example of sustainable cycles of materials in action. The works implemented not only repaired or refurbished the area in question but also made qualitative improvements in terms of aesthetic designs as well as functionality. The survey outcome from 96 respondents, including students, employees, and visitors, the survey employed a Likert scale (1–5) to assess aesthetic attractiveness, user comfort, and professional skill enhancement, indicated an increase in aesthetic attractiveness of 77% as perceived after implementation.

Every project site was marked by a design that clearly showed some kind of legacy of past material usage, symbolizing environmental and resource sustainability. This technique allowed each space to have a unique identity, something that was not repeated by the homogeneous and identity-less nature of factory-manufactured products. The above points prove that there is no exclusion between sustainability and aesthetic value, but instead they can complement each other. In terms of educational theory, the project delivered more than 1200 h of educator-led work in a project-based model of learning. There was a huge gap between the theoretical knowledge and the actual application. There was engagement for the learners in each level of the project process: conceptual (15%), material analysis (20%), fabrication and implementation (45%), and final project (20%). In this endeavor, the learners acquired technical expertise associated with material science, structures, and sustainable production, as well as soft skills such as teamwork, time management, and innovative problem-solving.

### The long-term implications of the interventions

The potential for long-term impacts on student performance and institutional sustainability is:

1. Student Performance: Participating students benefit from the hands-on experiences of upcycling as well as the improved learning spaces, as the gap between theory and practice is narrowed. In the long term, this is bound to improve student performance as:

- Technical skills in the areas of material handling, design, and sustainability.
- Soft skills like teamwork, problem-solving, and time management.
- Cognitive and environmental awareness, promoting responsible decision-making.

2. Institutional Sustainability: The inclusion of recycled materials, energy-efficient lighting, and environmentally conscious design will encourage sustainability on campus. In terms of benefits:

- Reduced waste generation and carbon footprint.
- Establishment of replicable models for other departments and campuses.
- Developed stronger institutional identity in terms of environmental stewardship.

### Project challenges

The key challenges encountered during project implementation and the strategies used to address them are:

1. Interdisciplinary coordination: The coordination effort between architecture, electrical, art education, and engineering necessitates planning. This was achieved through coordination meetings and clarity of roles and responsibilities.
2. Material sourcing and management - this was a challenge related to the availability of adequate materials for recycling from the campus waste materials. This was addressed through inventory tracking.
3. Minimizing Disruption to Academic Activities: The process of carrying out the interventions without halting the processes in the academic activities was handled by working during off-hours and creating alternative learning spaces for students.
4. Participant Engagement: This issue of maintaining hands-on participation was resolved by the inclusion of project-based learning sessions within the activities.

### Limitations of the study

Despite the encouraging outcomes of the proposed interventions, several limitations should be acknowledged.

1. The study was conducted within a single institutional environment characterized by specific waste streams and operational conditions. Consequently, the findings may not be directly generalizable to other educational institutions or settings with different infrastructural, behavioral, or regulatory contexts.
2. The projected lifespan of the implemented solutions, estimated at 5–8 years, is derived from material specifications and design assumptions. Actual long-term performance, degradation behavior, and maintenance requirements remain uncertain and can only be validated through extended operational monitoring.
3. The economic evaluation was limited to non-commercial labor inputs, as all activities were carried out by students, academic staff, and faculty members. Accordingly, the reported cost-effectiveness may not accurately reflect scenarios involving professional labor or large-scale commercial deployment.
4. The absence of designated control spaces limits the ability to fully isolate the observed improvements from other simultaneous institutional initiatives or environmental changes occurring at the campus level during the study period.
5. The lack of control spaces prevents isolating the improvements on one front from other concurrent and simultaneous measures or changes instituted in other aspects of the overall institution during the study period.

### Future work

Based on the identified limitations, different research directions can be considered to improve the research robustness in the future.

1. In the future, the implementation should be extended to various institutions, where diverse waste profiles and operational setups can be considered to increase the generalization and transferability of the study. Comparative analyses can also be incorporated in various educational and non-educational environments to further establish the applicability of the proposed method.
2. Long-term monitoring programs are also critical in estimating the long-term durability, deterioration characteristics, and lifespan of implemented solutions within actual operational environments, thus being able to give accurate estimates on lifespan.
3. Techno-economic analyses would also need to be conducted with the inclusion of commercial labor rates and market-based material costs to ensure the financial viability of such a scenario.
4. Future designs should incorporate appropriate control spaces or baseline reference areas to more rigorously isolate intervention effects, enhancing our causal inferences of improved performance.

## Conclusions

It becomes clear from this research that the idea of waste and the environment as mere concerns of waste management in the ecosystem could be repositioned to form the basis of a valuable resource that could be leveraged to improve the learning environments. It was made feasible through the application of an upcycling model that enabled the diversion of a significant amount of waste paper, metal, plastic, and wood that would otherwise have been sent to landfill. This approach contributed to a noticeable reduction in carbon emissions expressed as CO₂e. Based on surveys conducted before and after the implementation process, participants reported clear improvements in the aesthetic value of the four key environments within the university setting. The project also generated extensive collaborative work hours distributed across idea development, material analysis, fabrication, and evaluation/installation phases. It helped in encouraging the other departments and visitors to the university to access the idea of participating in the program so that it could be replicated. From the academic viewpoint, it becomes obvious that the program was able to achieve multiple objectives, including the development of the environment and the deployment of it as being effective in the professional upscaling of the participating members. It is advised that the program be applied in the same manner in the university setting and that permanent recycling programs be conducted in the form of participatory upcycling activities in the proposed environments to achieve the required objectives. It becomes essential that the impacts related to waste in the lifecycle reduction and the impacts of the improved environments be quantified in the future to assess the impacts on the performance of the university students. It could be evidenced that the proposed program could be applied as the basis of multiple Sustainable Development Goals and that it has the ability to form the basis of multiple tangible changes in the university environments. Future work could examine the long-term effects of participatory upcycling on student learning, environmental awareness, and professional skills, as well as the scalability of such interventions across other campuses. Practically, universities are encouraged to adopt ongoing upcycling programs, establish guidelines for managing campus waste, and implement replicable design frameworks that integrate aesthetic, environmental, and educational benefits for sustainable learning environments.

## Data Availability

All data generated or analyzed during this study are included in this published article. You can contact Khadija Shakra in case of requesting study data. this email: [Khadija.Shakra@techedu.bsu.edu.eg](mailto: Khadija.Shakra@techedu.bsu.edu.eg).
